# Hydrogen peroxide-triggered gene silencing in mammalian cells through boronated antisense oligonucleotides†
†Electronic supplementary information (ESI) available: Full experimental details, ^1^H, ^13^C and ^31^P spectra of all new compounds, HPLC charts, ESI-MS spectra of all new ODNs and representative UV melting data. See DOI: 10.1039/c7sc04318j


**DOI:** 10.1039/c7sc04318j

**Published:** 2017-12-06

**Authors:** Shohei Mori, Kunihiko Morihiro, Takumi Okuda, Yuuya Kasahara, Satoshi Obika

**Affiliations:** a Graduate School of Pharmaceutical Sciences , Osaka University , 1-6 Yamadaoka , Suita , Osaka 565-0871 , Japan . Email: morihiro@bioorg.rcast.u-tokyo.ac.jp ; Email: obika@phs.osaka-u.ac.jp; b National Institutes of Biomedical Innovation, Health and Nutrition (NIBIOHN) , 7-6-8 Saito-Asagi , Ibaraki , Osaka 567-0085 , Japan

## Abstract

Hydrogen peroxide (H_2_O_2_) is a reactive oxygen species (ROS) involved in various diseases, including neurodegeneration, diabetes, and cancer. Here, we introduce a new approach to use H_2_O_2_ to modulate specific gene expression in mammalian cells. H_2_O_2_-responsive nucleoside analogues, in which the Watson–Crick faces of the nucleobases are caged by arylboronate moieties, were synthesized. One of these analogues, boronated thymidine (**dT^B^**), was incorporated into oligodeoxynucleotides (ODNs) using an automated DNA synthesizer. The hybridization ability of this boronated ODN to complementary RNA was clearly switched in the off-to-on direction upon H_2_O_2_ addition. Furthermore, we demonstrated H_2_O_2_-triggered gene silencing in mammalian cells using antisense oligonucleotides (ASOs) modified with **dT^B^**. Our approach can be used for the regulation of any gene of interest by the sequence design of boronated ASOs and will contribute to the development of targeted disease therapeutics.

## Abstract 2

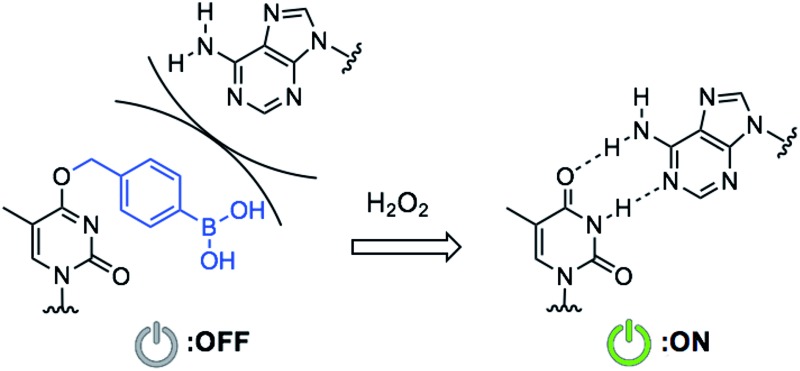
Arylboronic acid-modified antisense oligonucleotides enable hydrogen peroxide induced gene silencing in mammalian cells.

## Introduction

Nucleic acids are fundamental molecules for biological processes as they are responsible for the storage, regulation, and expression of genetic information. Modulating the biological activities of nucleic acids is of value for therapeutic and diagnostic applications. Antisense oligonucleotides (ASOs) are among the most promising oligonucleotides for the down-regulation of specific genes.^1^ ASOs hybridize with complementary mRNA to inhibit translation and reduce specific gene expression though the RNAse H degradation process or to modulate mRNA splicing pathways. Several antisense drugs have been approved by the US FDA in recent years.^2^–^5^ However, although ASOs are promising tools to control specific gene expression, a considerable drawback of the technology is the inability to regulate the activity of ASOs in a designated manner. If the activities of ASOs could be strictly controlled, as required for therapeutic use, their dosage could be reduced while maintaining their efficiency, and lesion specific knockdown of the target gene would be realized, reducing the off-target effect. Photo-caging technology represents an effective means to the development of the spatial and temporal control of ASO activity.^6^–^16^ In particular, nucleobase-caged ASOs have been systematically investigated, thus facilitating the manipulation of biological phenomena by photo-irradiation.^17^–^22^ However, the use of other stimuli, such as small molecule-responsive caged ASOs, remain largely unexplored.^23^ The chemical environment in our body, as well as inside cells, differs in space and over time. The ability of caged nucleic acids to alter their properties under a specific biomarker in a lesion would provide new options for nucleic acid therapeutics.

**Fig. 1:**
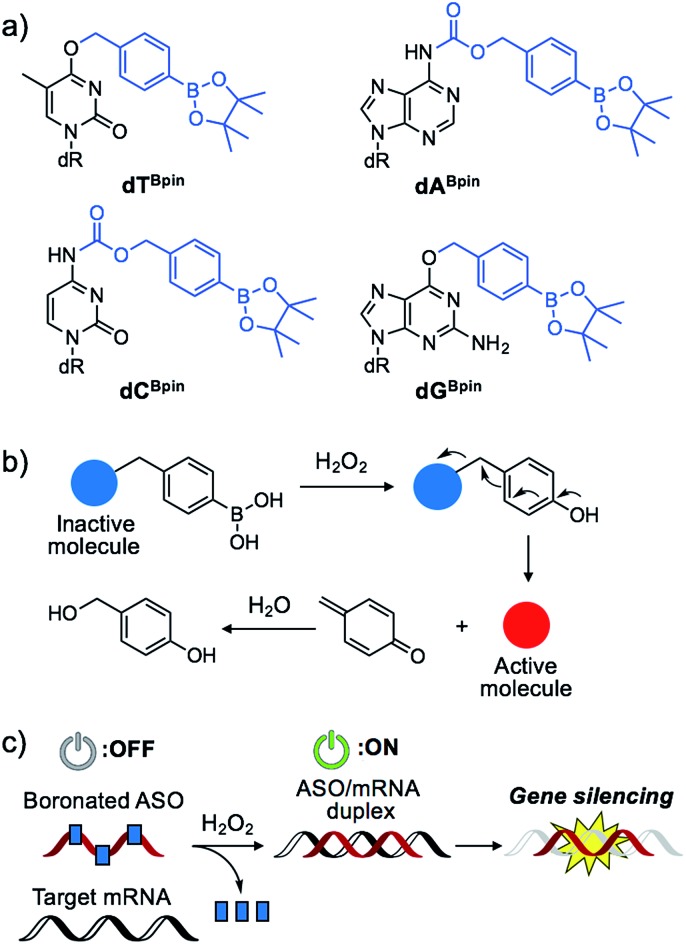
(a) Chemical structure of boronated 2′-deoxyribonucleoside analogues synthesized in this study. (b) Schematic of H_2_O_2_-triggered molecule activation. (c) Gene silencing triggered by H_2_O_2_ through inactive boronated ASOs.

Some diseased cells, such as inflamed cells^24^ and cancer cells,^25^ exhibit elevated intrinsic oxidative stress, and the amounts of reactive oxygen species (ROS) are increased. Thus, the development of molecular probes toward ROS is one of the best intriguing research fields.^26^ Hydrogen peroxide (H_2_O_2_) is an ROS that exhibits good reactivity and is present in significant concentrations *in vivo*, and is thus an ideal candidate as a biomarker of tumours. Compared with normal cells, cancer cells have increased levels of H_2_O_2_ of up to 0.5 nmol/10^4^ cells per h.^25^ There is therefore great interest in developing ASOs that are activated by excess H_2_O_2_. We designed a series of boronated nucleosides (**dT^Bpin^**, **dA^Bpin^**, **dC^Bpin^** and **dG^Bpin^**) for the H_2_O_2_-triggered activation of ASOs (Fig. 1a). Arylboronic acids and their esters can effectively temporally mask their molecular activity. They are oxidized to phenol derivatives by H_2_O_2_ and subsequent 1,6-elimination releases the active molecule (Fig. 1b). Boronated small molecules have been used for the fluorescent detection of H_2_O_2_,^27^ selective gene activation,^28^ and as cancer therapeutics.^29^–^31^ To adapt H_2_O_2_-responsive small molecule technology to ASOs, we masked the Watson–Crick face of the nucleobase with pinacol borane. The sterically hindered arylboronate groups will interfere with duplex formation between the ASO and target mRNA. In the presence of H_2_O_2_, the boronate group will be swiftly removed, resulting in gene silencing through hybridization of the ASO to the target mRNA (Fig. 1c).

## Results and discussion

### Synthesis of boronated nucleosides

**Scheme 1:**
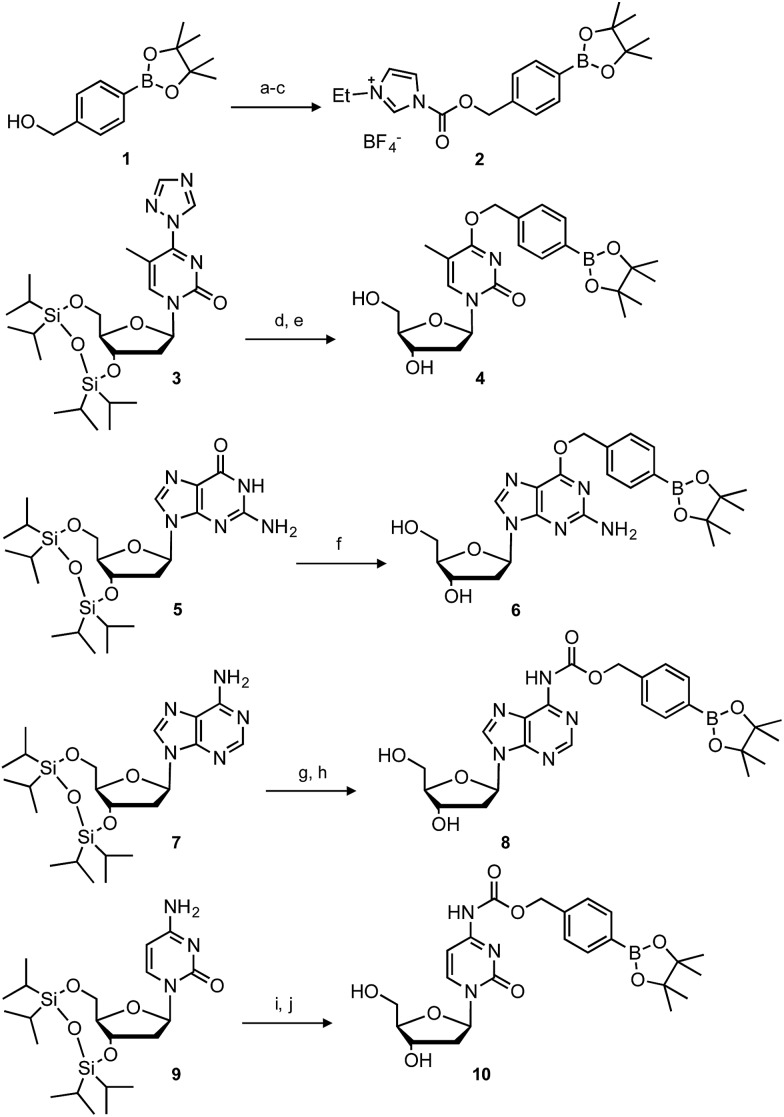
Synthesis of boronated nucleoside analogues. Conditions: (a) triphosgene, Na_2_CO_3_, toluene, rt; (b) imidazole, toluene, rt, 87% over 2 steps; (c) Et_3_OBF_4_, DCM, rt; (d) **1**, DBU, MeCN, rt, 98%; (e) TBAF, THF, 0 °C, 72%; (f) **1**, DIAD, PPh_3_, 1,4-dioxane, rt, then TBAF, 10%; (g) **2**, DCM, 0 °C to rt, 91%; (h) TASF, DMF, 0 °C, 62%; (i) **2**, DCM, 0 °C to rt, 92%; (j) HF-pyridine, 60 °C, 52%.

The synthesis of boronated nucleosides was started from suitably silylated sugars (Scheme 1). The H_2_O_2_ responsive pinacol borane moiety was introduced through a benzylic linker by S_N_Ar reaction of a triazolyl nucleoside derivative^32^ for **dT** and by Mitsunobu reaction for **dG**. The removal of TIPDS by TBAF afforded boronated nucleoside **dT^Bpin^** (**4**) and **dG^Bpin^** (**6**). In contrast, **dA^Bpin^** and **dC^Bpin^** have an arylboronate moiety linked *via* a carbamate spacer on their nucleobase. 4-(Hydroxymethyl) phenylboronic acid pinacol ester **1** was converted to the imidazole derivative in 2 steps. The imidazole was exposed to Meerwein reagent to prepare highly reactive imidazolium salt **2***in situ*, then reacted with TIPDS-protected dC or dA. TBAF treatment caused degradation of the carbamate moiety, and therefore we used HF-pyridine or TASF for silyl deprotection to afford **dA^Bpin^** (**8**) and **dC^Bpin^** (**10**) (Scheme 1).

### H_2_O_2_-decaging of boronated nucleosides

**Fig. 2:**
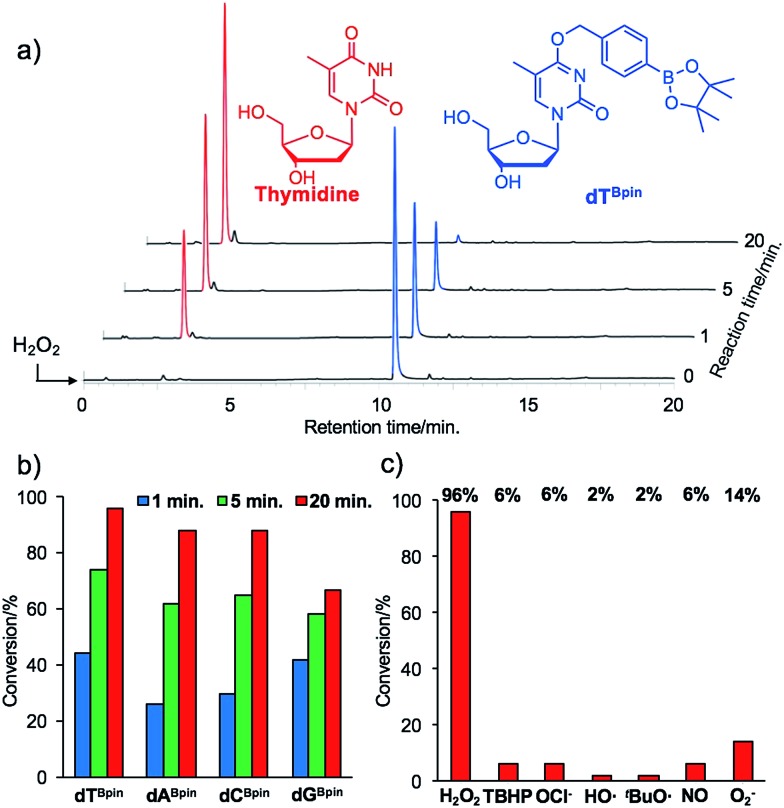
(a) HPLC chromatograms of **dT^Bpin^** after H_2_O_2_ addition at different time points. (b) Conversion rate of each boronated nucleoside analogue to the corresponding natural nucleoside. (c) H_2_O_2_-selectivity of **dT^Bpin^** toward other ROS.

With the caged nucleoside monomers in hand, we determined their conversion yields to the corresponding natural nucleosides by H_2_O_2_ treatment using HPLC analysis. Exposed to the equimolar equivalent of H_2_O_2_ in sodium phosphate buffer (pH = 7.2), virtually all of **dT^Bpin^** was converted to thymidine within 20 min (96% yield, Fig. 2a). Other boronated nucleosides were also promptly converted to the corresponding nucleosides in 67–88% yields (Fig. 2b and ESI†). The lower yield of **dG^Bpin^** was due to the generation of a side product, possibly the 8-oxo-dG analogue.^33^,^34^ Of the synthesized nucleoside analogues, **dT^Bpin^** showed the best reaction yield and kinetics for H_2_O_2_-decaging. To provide further insights into the decaging mechanism, we examined the inducible reactivity of **dT^Bpin^** toward other ROS, such as *tert*-butyl-hydroperoxide (TBHP), hypochlorite (ClO^–^), hydroxyl radical, *tert*-buthoxy radical, superoxide (O_2_^–^), and nitric oxide. We found that the decaging reaction of **dT^Bpin^** was highly selective for H_2_O_2_ over the other ROS (Fig. 2c). The selective reaction of **dT^Bpin^** with H_2_O_2_ is consistent with the observations of other groups.^28^–^31^ We also checked the reactivity of **dT^Bpin^** toward peroxynitrite (ONOO^–^), which is reported to be a potential oxidant of arylboronic acid moieties.^35^ However, in the same reaction conditions, almost no conversion to thymidine was observed (see HPLC chromatograms in ESI†). The half-life of ONOO^–^ in neutral buffer is extremely short^36^ and an equimolar equivalent of ONOO^–^ may not be sufficient for **dT^Bpin^** decaging.

### Boronated ODN synthesis

Although the introduction of arylboronate groups into oligodeoxynucleotides (ODNs) is very interesting due to their characteristic function,^37^,^38^ the direct incorporation of arylboronic acid moieties using solid phase synthesis is challenging because of their hygroscopicity. Wang and co-workers reported the synthesis of arylboronic acid-modified ODNs using a post-synthetic approach.^38^ However, this strategy limits the design of boronated ODNs. We predicted that phosphoramidites modified with pinacol borane would be less hygroscopic and could be applied to direct DNA synthesis.

**Fig. 3:**
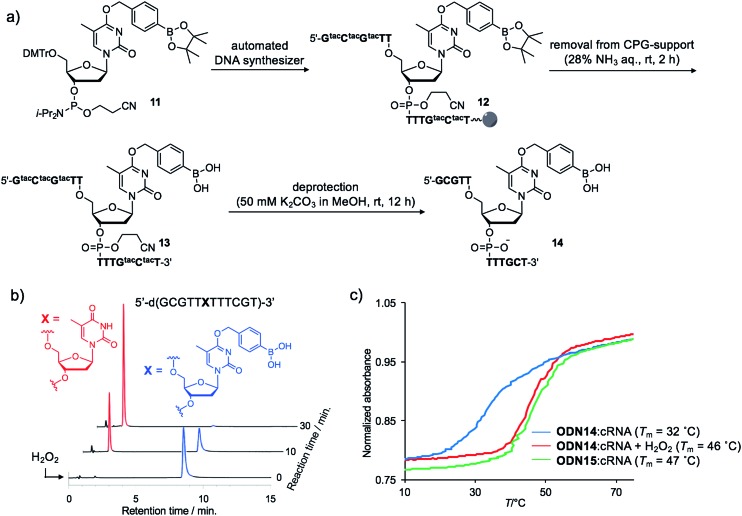
(a) Synthetic scheme of boronated ODN. tac = *tert*-butylphenoxyacetyl. (b) HPLC chromatograms of **ODN14** after H_2_O_2_ addition at different time points. (c) Thermal melting curves of the duplex formed between **ODN14** and complementary RNA in the presence or absence of H_2_O_2_.

We incorporated **dT^Bpin^** into ODN to take into account both the decaging efficiency and stability of the ODN under DNA synthesis conditions. Fortunately, phosphoramidite **11**, prepared from **dT^Bpin^** nucleoside **4** (see ESI† for the synthesis), could be incorporated into the ODN using standard solid phase synthesis procedures. After cleavage from the solid support with ammonium hydroxide, the protecting groups were removed by exposure to 50 mM K_2_CO_3_ in methanol (Fig. 3a). Subsequent HPLC purification afforded highly pure **ODN14** in which the pinacol ester group of **dT^Bpin^** was hydrolysed (see MALDI-TOF and ESI MS analysis in ESI†). **ODN14** was observed as the dehydrated form on ESI MS that corresponds to Wang's report.^38^ To the best of our knowledge, this is the first example of the incorporation of the arylboronic acid group into an ODN without the need to use a post-synthetic approach.

### H_2_O_2_-activation of boronated ODN

Next, we analysed the H_2_O_2_ reactivity of **dT^B^**-modified **ODN14** by HPLC (Fig. 3b). When **ODN14** was exposed to an excess amount of H_2_O_2_ at rt, the **ODN14** peak at 8.8 min completely disappeared and another peak appeared within 30 min in the HPLC profile. The resulting ODN, with a retention time of 2.0 min, was shown by MALDI-TOF MS spectrometry to be **ODN14** in which the boronic acid moiety of **dT^B^** was absent (see MALDI-TOF MS analysis in SI). The oxidation of **dT^B^** by H_2_O_2_ remained clean and rapid.

The duplex-forming ability of **ODN14** toward its complementary RNA (cRNA) strand was measured in the presence and absence of H_2_O_2_. Under the H_2_O_2_-free condition, the modified duplex **ODN14**:cRNA (Fig. 3c: blue line) was significantly destabilized compared with the unmodified **ODN15**:cRNA duplex (Fig. 3c: green line) (Δ*T*_m_ = 14 °C). The stabilities of the base pairs formed between **T^Bpin^** and other nucleobases (G, U, and C) were also lower than the T:A base pair (Table S1†). These results indicate that the boronic acid moiety effectively inhibits complementary base pairing, probably due to steric hindrance. The *T*_m_ of **ODN14**:cRNA in the presence of H_2_O_2_ (Fig. 3c: red line) was comparable to that of **ODN15**:cRNA (Δ*T*_m_ = 1 °C). The mismatch discrimination ability of **ODN14** was not decreased by H_2_O_2_ addition (Table S1†). These excellent hybridization properties of **ODN14** were also seen toward complementary DNA (Table S2†). These results suggest that **dT^B^**-modified ODNs enable switching of their hybridization to complementary strand in the off-to-on direction upon H_2_O_2_ treatment.

### H_2_O_2_-triggered gene silencing in mammalian cells

**Fig. 4:**
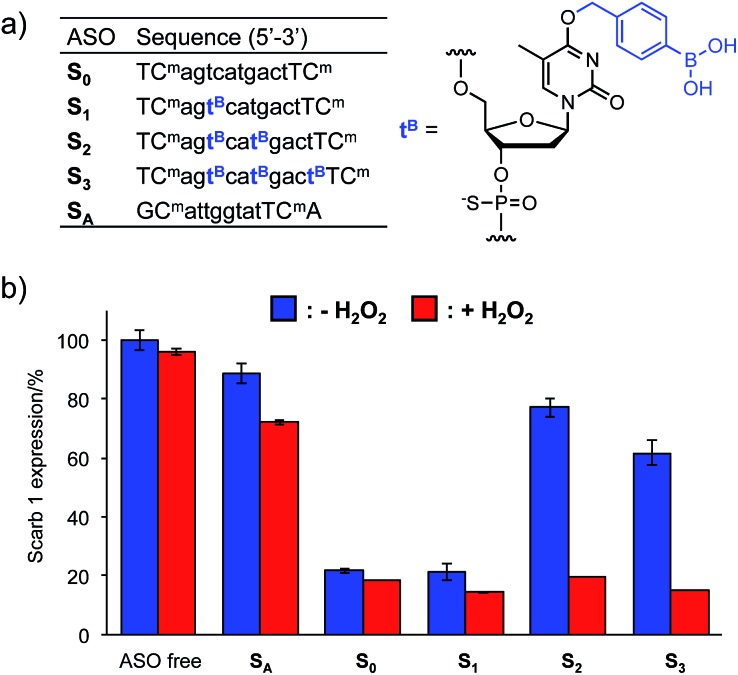
(a) Sequences of ASOs. n = DNA, N = LNA (C^m^ = LNA-5-Me-cytidine), all internucleosidic linkages are phosphorothioated. (b) Intercellular gene silencing triggered by H_2_O_2_ using boronate ASOs. Three independent experiments were averaged and the error bars represent standard deviation.

The *T*_m_ results prompted us to use the H_2_O_2_-responsive caged ODNs in biological systems. We incorporated **dT^B^** into the gap region of ASO targeting *Mus Scarb1* mRNA (Fig. 4a). Scarb1 has been implicated in several tumours,^39^ and targeted ASOs may provide a novel cancer therapeutic approach. **S_0_** includes phosphorothioate and locked nucleic acid (LNA) modifications to increase nuclease resistance and affinity toward the target mRNA, respectively. To determine the effect of boronate caging groups on antisense potency, we synthesized a set of three ASOs **S_1_–S_3_** containing 1–3 **dT^B^** units. Hepa-1c1c7 cells, a line derived from mice hepatoma, were treated with the ASOs by simple addition to the medium with CaCl_2_ (ref. 40) and incubated for 48 h in the absence or presence of H_2_O_2_ (10 μM). *Scarb1* mRNA levels were then quantified using qRT-PCR (Fig. 4b). Under the H_2_O_2_ free condition, the knockdown efficiency of target mRNA by **S_1_** was almost the same as that by **S_0_** (positive control). On the other hand, **S_2_** and **S_3_** showed low antisense efficiency, similar to **S_A_** (negative control). This result indicates that at least two **dT^B^** units are necessary to mask the gene silencing potency of the ASO. In contrast, when exposed to 10 μM H_2_O_2_, the antisense efficiencies of **S_2_** and **S_3_** are comparable to that of **S_0_**. The negligible change in gene silencing upon H_2_O_2_ treatment without ASO indicates that 10 μM H_2_O_2_ does not affect the expression level of *Mus Scarb1* mRNA. However, **S_A_** showed about 15% lower mRNA expression after H_2_O_2_ treatment, suggesting the combination of the modified oligonucleotide and H_2_O_2_ may affect the *Scarb1* mRNA expression level. Targeting other mRNAs or applying other chemical modifications could help to obtain further insights for this phenomenon. Taken together, these results clearly demonstrate that the ASOs **S_2_** and **S_3_** enable excellent off-to-on switching of gene silencing in mammalian cells triggered by H_2_O_2_.

## Conclusions

We designed and synthesized arylboronate-modified nucleosides which are promptly decaged and converted to natural nucleosides by H_2_O_2_. The boronated thymidine **dT^Bpin^** phophoramidite could be directly used in automated DNA synthesis and a **T^B^**:A base pair in the duplex could be employed as a temporary mismatch until H_2_O_2_ addition. Moreover, **dT^B^**-modified ASOs were demonstrated to be H_2_O_2_ switches that can control target mRNA expression. H_2_O_2_ is known to be involved in various diseases such as cancer, neurodegeneration,^41^ and diabetes.^42^ Our results indicate that boronated ASOs are promising tools to control target gene expression in a designated manner and as a prodrug-type nucleic acid therapeutic for selective disease treatment. Furthermore, the design of boronated nucleotides can be easily adapted to other nucleic acid agents such as aptamers, triplex-forming oligonucleotides (TFOs), and small interfering RNAs (siRNAs).

## Experimental

### General

Reagents and solvents were purchased from commercial suppliers and used without purification unless otherwise specified. All experiments involving air and/or moisture sensitive compounds were carried out under an Ar atmosphere. All reactions were monitored with analytical TLC (Merck Kieselgel 60 F254; Merck, Darmstadt, Germany). Flash column chromatography was carried out using EPCLC-W-Prep 2XY (YAMAZEN, Osaka, Japan). Physical data were measured as follows. NMR spectra were recorded on a JNM-ECS-400 spectrometer (JEOL, Tokyo, Japan) using CDCl_3_ or DMSO-*d*_6_ as the solvent with tetramethylsilane as an internal standard. IR spectra were recorded on a FT/IR-4200 spectrophotometer (JASCO, Tokyo, Japan). Optical rotations were recorded on a JASCO P-2200 instrument. FAB mass spectra were measured using a JEOL JIM-700 mass spectrometer. Solid-phase ODN synthesis was performed using an nS-8 II oligonucleotide synthesizer (GeneDesign, Osaka, Japan). MALDI-TOF mass spectra were recorded on an ultrafleXtreme mass spectrometer (Bruker Daltonics, Billerica, MA, USA). ESI mass spectra were recorded on a Xevo G2-XS QTof (Waters, Milford, MA, USA). UV/vis absorption measurements and UV melting experiments were performed using a UV-1650PC UV-vis spectrophotometer equipped with a TMSPC-8 *T*_m_ analysis accessory (SHIMADZU, Kyoto, Japan).

### H_2_O_2_-decaging of boronated nucleosides

A reaction solution of boronated nucleoside (1 mM) and H_2_O_2_ (1 mM) in a DMSO-containing buffer (10 mM potassium phosphate, pH 7.2, 100 mM NaCl, and DMSO 5% v/v) was incubated at room temperature for the prescribed times and immediately subjected to reversed-phase HPLC analysis using MeCN in 0.1 M triethylammonium acetate buffer (pH 7.0). The conversion rate of boronated nucleoside was determined from the corresponding peak area monitored at 260 nm.

### Other ROS-decaging of **dT^Bpin^**

A reaction solution of boronated nucleoside (1 mM) and reactive oxygen species (1 mM) in a DMSO-containing buffer (10 mM potassium phosphate, pH 7.2, 100 mM NaCl, and DMSO 5% v/v) was incubated at room temperature for 12 h and immediately subjected to reversed-phase HPLC analysis using MeCN in 0.1 M triethylammonium acetate buffer (pH 7.0). H_2_O_2_, *tert*-butylhydroperoxide (TBHP), and hypochlorite (NaOCl) were delivered from 30%, 70%, and 10% aqueous solutions respectively. Hydroxyl radical (HO˙) and *tert*-butoxy radical (^*t*^BuO˙) were generated by the reaction of 5 mM (NH_4_)_2_Fe(SO_4_)_2_, 10 mM EDTA with 1 mM H_2_O_2_ or TBHP, respectively. Nitric oxide (NO) was generated from PROLI NONOate. Superoxide (O_2_^–^) was produced by xanthine oxidase (4.5 × 10^–3^ mg/100 μL) in the presence of hypoxanthine (2 mM) and catalase (0.4 mg mL^–1^). Peroxynitrite (ONOO^–^) was delivered from NaOH aqueous solution and the concentration of ONOO^–^ was determined using the absorption at 300 nm (*ε* = 1670 M^–1^ cm^–1^). The solution was diluted with phosphate buffer and used immediately. The conversion rate of boronated nucleoside was determined from the corresponding peak area monitored at 260 nm.

### ODN synthesis

Solid-phase ODN synthesis was performed using commercially available reagents and phosphoramidites. **dT^Bpin^** phosphoramidite was chemically synthesized as described (Scheme S1†). ODNs were synthesized (with trityl-on) on a 500 Å CPG solid support column (1 μmol scale) using 5-ethylthio-1*H*-tetrazole (0.25 M in MeCN) as the activator and 0.05 M ((dimethylaminomethylidene)amino)-3*H*-1,2,4-dithiazaoline-3-thione (DDTT) in pyridine/MeCN (1 : 1, v/v) for thiolation. The standard synthesis cycle was used for assembly of the reagents and synthesis of the oligonucleotides, except that the coupling time was extended to 5 min for LNA monomers. Cleavage from the solid support and deprotection were accomplished with 28% w/w NH_4_OH aqueous solution at room temperature for 2 h and potassium carbonate (0.05 M in methanol solution) at room temperature for 12 h, respectively. The crude ODNs were purified on a Sep Pack column (Waters) followed by RP-HPLC on an XBridge™ OST C18 column, 2.5 μm, 10 × 50 mm (Waters) using MeCN in 0.1 M triethylammonium acetate buffer (pH 7.2). The purified ODNs were quantified by UV absorbance at 260 nm and confirmed by MALDI-TOF or ESI mass spectrometry. In ESI MS measurement, boronated ODN and ASO were detected as the dehydrated form, which is consistent with the observation of Wang's group.^38^

### H_2_O_2_-decaging of boronated ODN

A reaction solution of **ODN14** (4 μM) and H_2_O_2_ (1 mM) in a buffer (10 mM potassium phosphate, pH 7.2, 100 mM NaCl) was incubated at room temperature for the prescribed times and immediately subjected to reversed-phase HPLC analysis using MeCN in 0.1 M triethylammonium acetate buffer (pH 7.0).

### UV melting experiments

Melting temperatures (*T*_m_) were determined by measuring the change in absorbance at 260 nm as a function of temperature using a SHIMADZU UV-vis UV-1650PC spectrophotometer equipped with a TMSPC-8 *T*_m_ analysis accessory. The melting samples were denatured at 100 °C and annealed slowly to room temperature. Absorbance was recorded in the forward and reverse direction at temperatures between 5 to 90 °C at a rate of 0.5 °C min^–1^.

### Cell culture

Hepa-1c1c7 cells were obtained from the European Collection of Authenticated Cell Cultures (ECACC; Salisbury, UK). The cell line was maintained at 37 °C and 5% CO_2_ in Dulbecco's Modified Eagle's Medium (DMEM; Nacalai Tesque, Kyoto, Japan) supplemented with 10% heat-inactivated foetal bovine serum (FBS) and antibiotics.

### Evaluation of the *in vitro* activity of **dT^B^**-modified ASOs in the absence or presence of H_2_O_2_

Hepa-1c1c7 cells were seeded at 5.0 × 10^3^ cells per well in 96-well plates (Corning, New York, NY, US) containing 10% FBS/DMEM. After 24 h, 100 nM ASO was added and the cells were cultured in medium supplemented with 9 mM CaCl_2_ in the absence or presence of H_2_O_2_ (10 μM). After 48 h, total RNA was isolated using a QIAGEN RNeasy kit (QIAGEN, Valencia, CA, USA). Reduction of target mRNA expression was determined by real time qRT-PCR using a One Step SYBR PrimeScript PLUS RT-PCR kit (Takara Bio Inc., Kusatsu, Japan) and analysed with a StepOnePlus system (Applied Biosystems; Foster City, CA, USA). The primers used in this study were specific for the mouse *Scarb1* gene (forward: 5′-TGACAACGACACCGTGTCCT-3′; reverse: 5′-ATGCGACTTGTCAGGCTGG-3′) and for the mouse *GAPDH* gene (forward: 5′-TGCACCACCAACTGCTTAG-3′; reverse: 5′-GATGCAGGGATGATGTTC-3′). The level of target (*Scarb1*) gene expression was normalized to that of *GAPDH* in the absence of H_2_O_2_.

## Conflicts of interest

There are no conflicts to declare.

## Supplementary Material

Supplementary information
