# Supplementary material for: Hydrogen peroxide-triggered gene silencing in mammalian cells through boronated antisense oligonucleotides
Source: Chem Sci. 2017 Dec 6;9(5):1112–8. doi: 10.1039/c7sc04318j (PMC5875086; doi:10.1039/c7sc04318j)
Supplement: Supplementary file 1 [file SC-009-C7SC04318J-s001.pdf]

Electronic Supplementary Material (ESI) for Chemical Science.
This journal is © The Royal Society of Chemistry 2017

Supplementary Information

Hydrogen peroxide-triggered gene silencing in mammalian cells through boronated antisense oligonucleotides

Shohei Mori,[a] Kunihiko Morihiro,*[a,b†] Takumi Okuda,[a] Yuuya Kasahara,[a,b] and Satoshi Obika*[a,b]

[a] Graduate School of Pharmaceutical Sciences, Osaka University, 1-6 Yamadaoka, Suita, Osaka 565-0871, Japan

[b] National Institutes of Biomedical Innovation, Health and Nutrition (NIBIOHN), 7-6-8 Saito-Asagi, Ibaraki, Osaka 567-0085, Japan

[*] Corresponding authors. E-mails: morihiro@bioorg.rcast.u-tokyo.ac.jp (K.M.); obika@phs.osaka-u.ac.jp (S.O.)

[†] Present address: Department of Chemistry and Biotechnology, Graduate School of Engineering, The University of Tokyo

Contents

1. Synthesis of boronated nucleoside analogues and $\mathbf{dT^B}$ phosphoramidite S2

2. ^{1}H-, ^{13}C- and ^{31}P-NMR spectra of new compounds S10

3. H_2O_2-decaging of boronated nucleosides S31

4. Peroxynitrite ($ONOO^-$)-decaging of $\mathbf{dT^{Bpin}}$ S32

5. ESI and MALDI-TOF MS analysis of $\mathbf{dT^B}$-modified ODNs S33

6. UV melting experiments of duplexes containing $\mathbf{dT^B}$ without or with H_2O_2 S43

7. Reference S44

1. Synthesis of boronated nucleoside analogues and dT^B phosphoramidite

1-1. 4-(4,4,5,5-Tetramethyl-1,3,2-dioxaborolan-2-yl)1*H*-imidazole-1-carboxylate (17)

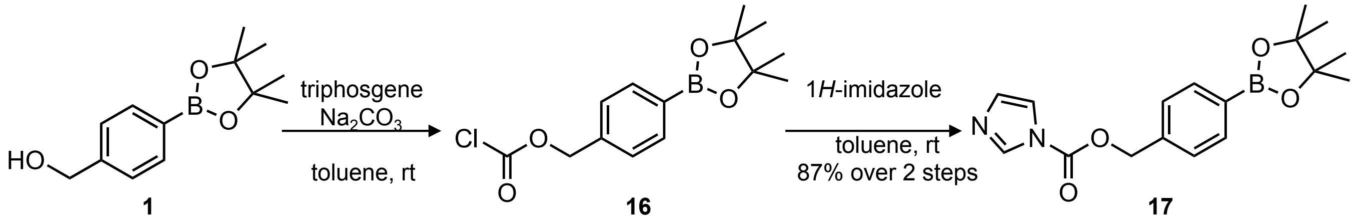

Na_2CO_3 (3.5 g, 33.0 mmol) was placed in a flame dried round-bottom flask and triphosgene (2.2 g, 7.4 mmol) in toluene (15 mL) was added at 0 ˚C. After stirring for 1 h at 0 °C, benzyl alcohol **1** (0.87 g, 3.7 mmol) in toluene (5 mL) was added and stirred for 6 h at room temperature. The insoluble residues were filtered off through a Celite pad. After the solvent was removed in vacuo, the resulting chloroformate **16**[S1] was used without further purification. Chloroformate **16** (1.09 g, 3.70 mmol) was dissolved in dry toluene (20 mL) and 1*H*-imidazole (1.00 g, 14.8 mmol) was added at room temperature. The reaction mixture was stirred for 4 h at room temperature and partitioned between AcOEt and H_2O. The separated organic layer was washed with brine, dried over Na_2SO_4 and concentrated in vacuo. The residue was purified by a silica gel column chromatography, eluted with hexane/AcOEt (2:1) to give compound **17** (1.06 g, 87% over two steps) as a white foam. IR (KBr): ν 3130 (Ar C-H), 1762 (C=O), 1615 (C=N) cm^{-1}; ^1H-NMR (300 MHz, $CDCl_3$): δ 8.15 (1H, s), 7.87 (2H, d, J = 6.0 Hz), 7.48-7.41 (3H, m), 7.05 (1H, s), 5.04 (2H, s), 1.34(12H, s); ^{13}C-NMR (100 MHz, $CDCl_3$): δ 148.2, 136.8, 136.5, 134.9, 130.4, 127.4, 116.8, 83.6, 69.3, 24.6; FAB-LRMS m/z = 329 (MH^+); FAB-HRMS calcd for $C_{17}H_{22}N_2O_4B$ = 329.1676, found 329.1668.

1-2. 3-Ethyl-1-(((4-(4,4,5,5-Tetramethyl-1,3,2-dioxaborolan-2-yl)benzyl)oxy)carbonyl-1*H*-imidazol-3-ium tetrafluoroborate (2)

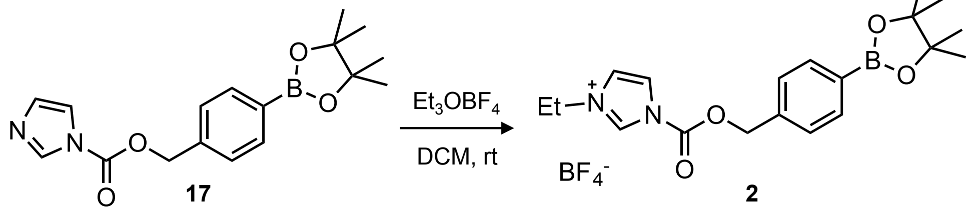

Compound **17** (1.21 g, 3.70 mmol) was dissolved in dry DCM (40 mL) and Et_3OBF_4 (669 mg, 3.52 mmol) was added at room temperature. The reaction mixture was stirred for 16 h at room temperature and the resulting imidazolium salt **2** was used without further purification.

1-3. (4-(4,4,5,5-Tetramethyl-1,3,2-dioxaborolan-2-yl)benzyl) -3,5-*O*-(1,1,3,3-tetraisopropyldisiloxane-1,3,diyl)-2'-deoxy thymidine (18)

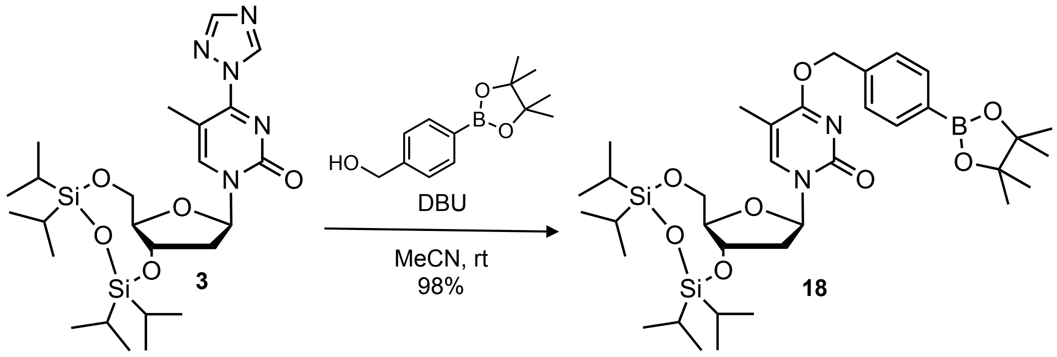

Compound **3** (160 mg, 0.297 mmol) was dissolved in dry MeCN (5 mL) and 2-(4-hydroxymethylphenyl)-4,4,5,5-tetramethyl-1,3,2-dioxaborolane (139 mg, 0.594 mmol) and DBU (89 μL, 0.594 mmol) were added at room temperature. The reaction mixture was stirred for 4 h at room temperature and the solvent was removed in vacuo. The residue was purified by a silica gel column chromatography, eluted with hexane/AcOEt (7:3) to give compound **18** (204 mg, 98%) as a white foam. IR (KBr): *v* 2943 (Ar C-H), 1670 (C=O), 1532 (C=N) cm^{-1}; $[\alpha]_D^{24}$ 25.0 (c 1.00, $CHCl_3$); ^{1}H-NMR (300 MHz, $CDCl_3$): δ 7.86-7.75 (3H, m), 7.39 (2H, dd, *J* = 12.0, 7.5 Hz), 6.05 (H, d, *J* = 6.5 Hz), 5.43 (2H, dd, *J* = 13.0, 16.0 Hz), 4.46-4.38 (1H, m), 4.19 (1H, d, *J* = 13.0 Hz), 4.03-4.00 (1H, m), 3.80-3.78 (1H, m), 2.62-2.52 (1H, m), 2.38-2.31 (1H, m), 1.99 (3H, s), 1.34 (12H, s), 1.14-0.95 (28H, m) ; ^{13}C-NMR (75 MHz, $CDCl_3$): δ 169.9, 155.4, 144.5, 139.4, 138.7, 134.7, 134.6, 126.9, 125.7, 103.8, 84.9, 84.8, 83.5, 83.4, 83.5, 83.4, 77.2, 68.3, 66.3, 64.4, 59.5, 39.6, 24.6, 17.2, 17.1, 17.0, 16.8, 16.7, 16.6, 13.2, 12.7, 12.4, 12.2, 12,1; MS (FAB) *m/z* 723 $[M+Na]^+$; HRMS (FAB): Calcd for $C_{35}H_{57}N_2O_8BSi_2Na$ $[M+Na]^+$: 723.3644. Found: 723.33651.

1-4. (4-(4,4,5,5-Tetramethyl-1,3,2-dioxaborolan-2-yl)benzyl)-2'-deoxy thymidine (4)

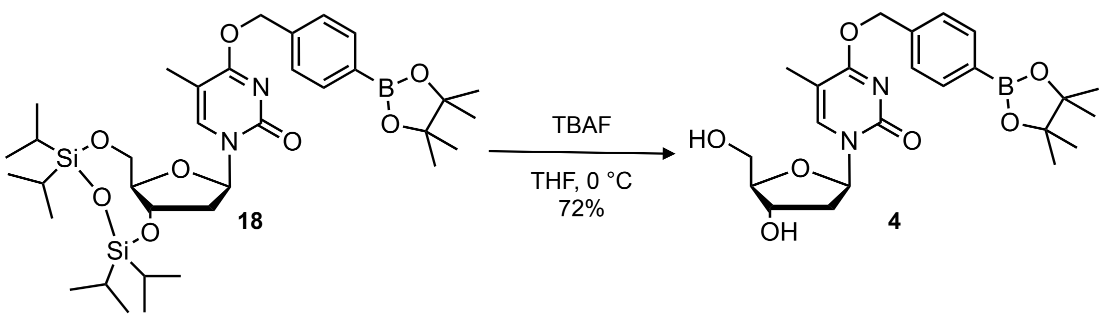

To a solution of **18** (160 mg, 0.227 mmol) in dry THF (2.5 mL) was added 1 M TBAF solution in THF (478 μL, 0.478 mmol) was added dropwise at 0 °C, and the reaction mixture was stirred for 10 min. and then concentrated in vacuo.

The residue was purified by a silica gel column chromatography, eluted with AcOEt/MeOH (97:3) to give **4** (78 mg, 72%) as a white foam. IR (KBr): *v* 3335 (-OH), 2977 (Ar C-H), 1752 (C=O), 1660 (C=N) cm^{-1}; $[\alpha]_D^{24}$ 33.5 (c 1.00, MeOH); ^{1}H-NMR (300 MHz, CD_3OD): δ 8.08 (1H, s), 7.64 (2H, d, *J* = 8.0 Hz), 7.32 (2H, d, *J* = 8.0 Hz), 6.14 (1H, dd, *J* = 6.0, 6.5 Hz), 5.31 (2H, s), 4.30-4.26 (1H, m), 3.88-3.84 (1H, m), 3.74 (1H, dd, *J* = 3.0, 12.0 Hz), 3.64 (1H, dd, *J* = 3.0, 12.0 Hz), 2.95-2.90 (1H, m), 2.34-2.28 (1H, m), 2.10-2.03 (1H, m), 1.89 (3H, s), 1.22 (12H, s); ^{13}C-NMR (75 MHz, CD_3OD): δ 171.7, 158.0, 142.4, 140.4, 135.9, 128.1, 106.5, 89.1, 87.9, 85.1, 79.5, 71.6, 69.6, 62.4, 54.0, 42.2, 27.1, 25.2, 21.0, 14.0, 12.3; MS (FAB) *m/z* 481 $[M+Na]^+$; HRMS (FAB): Calcd for $C_{23}H_{31}N_2O_7BNa$ $[M+Na]^+$: 481.2122. Found: 481.2126.

1-5. (6-(4,4,5,5-Tetramethyl-1,3,2-dioxaborolan-2-yl)benzyl)oxycarbonyl-2'-deoxy guanosine (6)

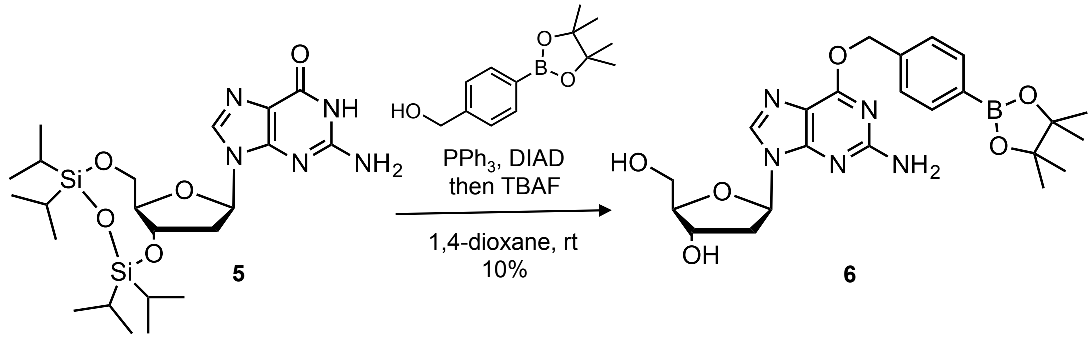

Compound **5** (300 mg, 0.585 mmol) was dissolved in dry 1,4-dioxane (6 mL) and Ph_3P (184 mg, 0.702 mmol), DIAD (138 µL, 0.702 mmol) and 2-(4-hydroxymethylphenyl)-4,4,5,5-tetramethyl-1,3,2-dioxaborolane (144 mg, 0.614 mmol) were added at room temperature. The reaction mixture was stirred for 4 h at room temperature and cooled in an ice bath. TBAF solution in THF (1 M, 1.23 mL, 1.23 mmol) was added dropwise at 0 ˚C and the resulting mixture was stirred for 10 min. The solvent was removed in vacuo and the residue was purified by a silica gel column chromatography, eluted with AcOEt/MeOH (19:1) to give compound **6** (28 mg, 10%) as a white foam. IR (KBr): *v* 3329 (-OH), 1585 (C=N) cm^{-1}; $[\alpha]_D^{24}$ −3.6 (c 1.00, MeOH); ^{1}H-NMR (300 MHz, CD_3OD): δ 8.03 (H, s), 7.91 (2H, s), 7.60 (2H, d, *J* = 8.0 Hz), 7.40 (2H, d, *J* = 8.0 Hz), 6.30 (1H, dd, *J* = 6.5, 9.0 Hz), 5.50 (2H, s), 4.58-4.51 (1H, m), 4.06-4.00 (1H, m), 3.86-3.67 (2H, m), 2.85-2.70 (1H, m), 2.38-2.27 (1H, m), 1.27-1.20 (12H, m); ^{13}C-NMR (75 MHz, CD_3OD): δ 180.1, 162.2, 161.4, 154.2, 140.0, 134.6, 128.3, 115.9, 89.7, 86.8, 79.5, 73.1, 69.4, 63.7, 47.4, 41.2, 24.1, 9.4, 9.3; MS (FAB) *m/z* 506 $[M+Na]^+$; HRMS (FAB): Calcd for $C_{23}H_{30}N_5O_6BNa$ $[M+Na]^+$: 506.3219. Found: 506.3224.

1-6. (6-(4,4,5,5-Tetramethyl-1,3,2-dioxaborolan-2-yl)benzyl)oxycarbonyl-3,5-*O*-(1,1,3,3-tetraisopropyldisiloxane-1,3,diyl)-2'-deoxy adenosine (19)

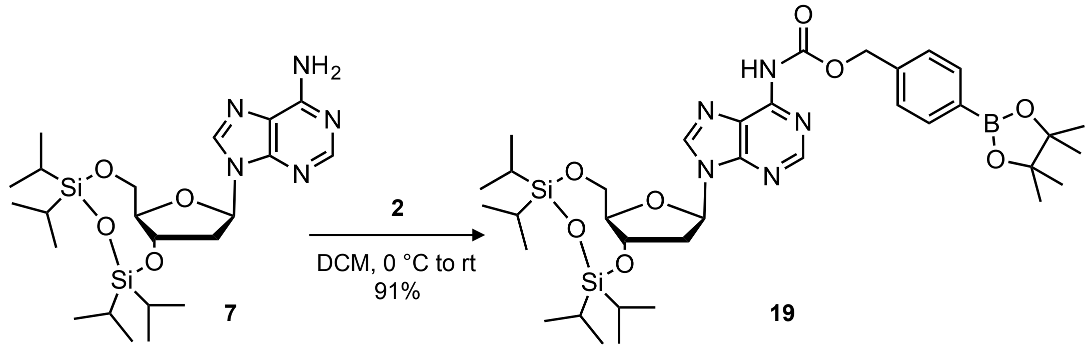

Compound **7** (124 mg, 0.25 mmol) was dissolved in dry DCM (10 mL) and compound **2** (444 mg, 1.00 mmol) in DCM (10 mL) was added at 0 °C. The reaction mixture was stirred for 24 h at room temperature and quenched by addition of saturated aqueous $NaHCO_3$. The resulting mixture was partitioned between DCM and H_2O. The separated organic layer was washed with brine, dried over Na_2SO_4 and concentrated in vacuo. The residue was purified by a silica gel column chromatography, eluted with hexane/AcOEt (4:1) to give compound **19** (172 mg, 91%) as a white foam. IR (KBr): *v*1758 (C=O), 1615 (C=N) cm^{-1}; $[\alpha]_D^{24}$ −23.4 (c 1.00, $CHCl_3$); ^{1}H-NMR (300 MHz, $CDCl_3$): δ 10.24 (1H, s), 8.74 (1H, s), 8.04 (1H, s), 7.81 (2H, d, *J* = 8.0 Hz), 7.39 (2H, d, *J* = 8.0 Hz), 6.00 (1H, d, *J* = 6.5 Hz), 5.36-5.23 (2H, m), 5.16-5.08 (1H, m), 4.03-3.98 (2H, m), 3.92-3.84 (1H, m), 2.81-2.56 (2H, m), 1.36 (12H, s), 1.13-1.02 (28H, m); ^{13}C-NMR (75 MHz, $CDCl_3$): δ 152.3, 151.1, 149.9, 149.5, 144.1, 141.9, 138.1, 134.8, 134.6, 127.6, 125.7, 122.4, 84.9, 83.6, 83.4, 83.3, 77.2, 70.0, 67.2, 64.4, 61.7, 39.4, 24.6(2), 24.5(9), 17.3, 17.0(9), 17.0(7), 17.0(6), 16.9, 16.8, 16.7, 16.6, 13.0, 12.8, 12.5, 12.2; MS (FAB) *m/z* 754 $[M+H]^+$; HRMS (FAB): Calcd for $C_{36}H_{57}N_5O_8BSi_2$ $[M+H]^+$: 754.3839. Found: 754.3846.

1-7. (6-(4,4,5,5-Tetramethyl-1,3,2-dioxaborolan-2-yl)benzyl)oxycarbonyl-2'-deoxy adenosine (8)

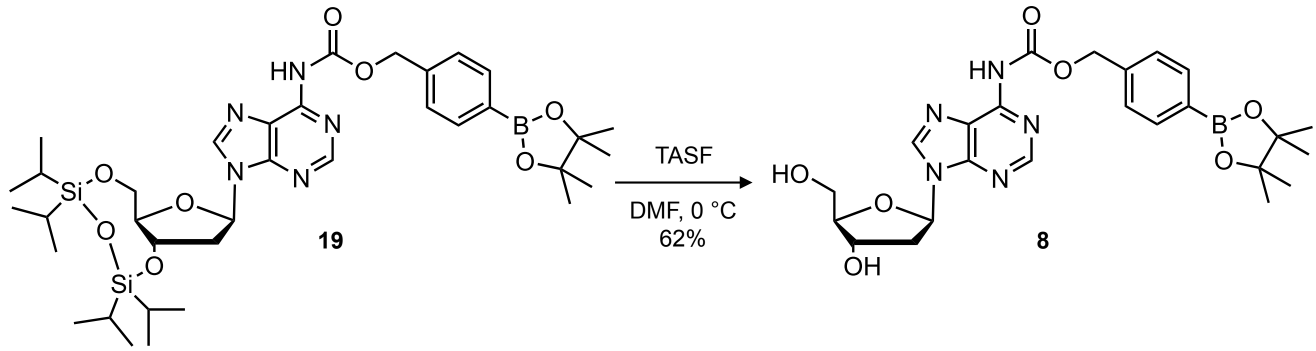

Compound **19** (1.19 g, 1.59 mmol) was dissolved in dry DMF (20 mL) and TASF (1.00 g, 3.63 mmol) was added at 0 °C. The reaction mixture was stirred for 1 h at 0 °C and partitioned between $CHCl_3$/2-propanol (3:1) and H_2O. The separated organic layer was concentrated in vacuo. The residue was purified by a silica gel column chromatography, eluted with $CHCl_3$/MeOH (19:1) to give compound **8** (520 mg, 62%) as a white foam. IR (KBr): *v* 3293 (-OH), 2977 (Ar C-H), 1757 (C=O), 1619 (C=N) cm^{-1}; $[\alpha]_D^{24}$ 1.2 (c 1.00, DMSO); ^{1}H-NMR (300 MHz, DMSO-*d*6): *δ* 10.80 (1H, brs), 8.66 (1H, s), 8.63 (1H, s), 7.68 (2H, d, *J* = 8.0 Hz), 7.46 (2H, d, *J* = 8.0 Hz), 6.44 (1H, dd, *J* = 6.5, 7.0 Hz), 5.23 (2H, s), 4.47-4.39 (1H, m), 3.91-3.84 (1H, m), 3.66-3.46 (2H, m), 2.82-2.70 (1H, m), 2.39-2.25 (1H, m), 1.29 (12H, s); ^{13}C-NMR (75 MHz, DMSO-*d*6): *δ* 152.1, 151.6, 151.5, 149.7, 142.8, 139.8, 134.5, 126.9, 123.8, 88.0, 83.7(5), 83.7(2), 70.7, 66.0, 61.6, 48.6, 25.5, 24.7; MS (FAB) *m/z* 512 $[M+H]^+$; HRMS (FAB): Calcd for $C_{24}H_{31}N_5O_7B$ $[M+H]^+$: 512.2317. Found: 512.2321.

1-8. (4-(4,4,5,5-Tetramethyl-1,3,2-dioxaborolan-2-yl)benzyl)oxycarbonyl-3,5-*O*-(1,1,3,3-tetraisopropyldisiloxane-1,3,diyl)-2'-deoxy cytidine (20)

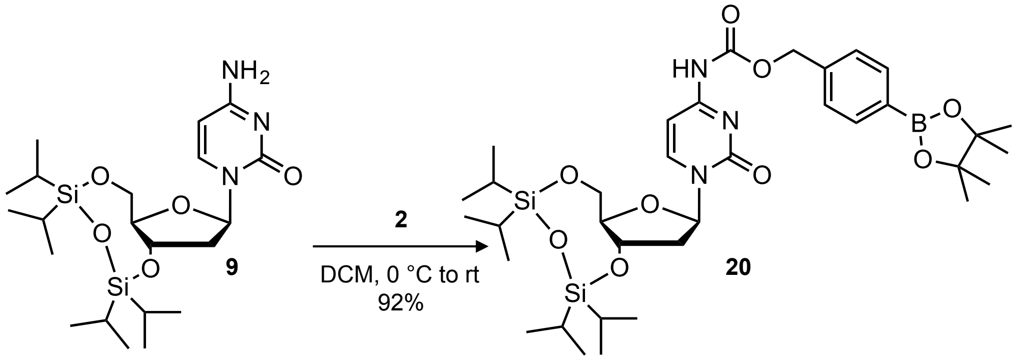

Compound **9** (416 mg, 0.88 mmol) was dissolved in dry DCM (10 mL) and compound **2** (1.56 g, 3.50 mmol) in DCM (10 mL) was added at 0 °C. The reaction mixture was stirred for 24 h at room temperature. and quenched by addition of saturated aqueous $NaHCO_3$. The resulting mixture was partitioned between DCM and H_2O. The separated organic layer was washed with brine, dried over Na_2SO_4 and concentrated in vacuo. The residue was purified by a silica gel column chromatography, eluted with hexane/AcOEt (4:1) to give compound **20** (593 mg, 92%) as a white foam. IR (KBr): *v* 3151 (Ar C-H), 1747 (C=O), 1622 (C=N) cm^{-1}; $[\alpha]_D^{24}$ 27.7 (c 1.00, $CHCl_3$); ^{1}H-NMR (300 MHz, $CDCl_3$): *δ* 8.80 (1H, drs), 8.22 (1H, d, *J* = 7.5 Hz), 7.81 (2H, d, *J* = 8.0 Hz), 7.37 (2H, d, *J* = 8.0 Hz), 7.23 (1H, d, *J* = 7.5 Hz), 6.03 (1H, d, *J* = 7.0 Hz), 5.21 (2H, s), 4.43-4.30 (1H, m), 4.21 (1H, d, *J* = 13.0 Hz), 4.02 (1H, dd, *J* = 3.0, 13.0 Hz), 3.81 (1H, d, *J* = 8.0 Hz), 2.63-2.49 (1H, m), 2.41-2.28 (1H, m), 1.34 (12H, s), 1.13-0.92 (28H, m); ^{13}C-NMR (75 MHz, $CDCl_3$): *δ* 162.4, 137.9, 135.0, 134.8, 134.7, 134.6, 126.9, 125.7, 85.3, 84.9, 83.5(4), 83.5(1), 83.4, 77.2, 67.3, 66.1, 59.5, 39.3, 24.6, 17.2, 17.1(6), 17.0(8), 17.0(1), 16.7(5), 16.6(9), 16.6(5), 16.6, 13.1, 12.7, 12.6, 12.1; FAB-LRMS m/z = 752

(MNa+); FAB-HRMS calcd for $C_{35}H_{56}O_9N_3BSi_2Na$= 752.3546, found 752.3553; MS (FAB) *m/z* 752 $[M+Na]^+$; HRMS (FAB): Calcd for $C_{35}H_{56}N_3O_9BSi_2Na$ $[M+Na]^+$: 752.3546. Found: 752.3553.

1-9. (4-(4,4,5, 5-Tetramethyl-1,3,2-dioxaborolan-2-yl)benzyl)oxycarbonyl-2'-deoxy cytidine (10)

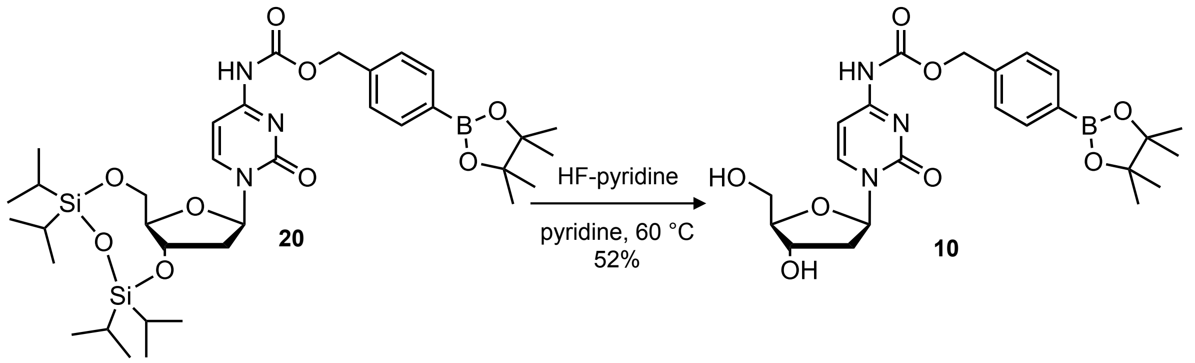

Compound **20** (1.99 g, 2.70 mmol) was dissolved in dry pyridine (20 mL) and HF-pyridine (ca 65% HF w/w. 604 μL, 10.8 mmol) was added at room temperature. The reaction mixture was stirred for 12 h at 60 °C and cooled to room temperature. The reaction was quenched by addition of solid $NaHCO_3$ and the insoluble residues were filtered off through a Celite pad. After the solvent was removed in vacuo, the residue was purified by a silica gel column chromatography, eluted with $CHCl_3$/MeOH (9:1) to give compound **10** (684 mg, 52%) as white foam. IR (KBr): *v* 3331 (-OH), 2979 (Ar C-H), 1750 (C=O), 1651 (C=N) cm^{-1}; $[\alpha]_D^{24}$ 55.7 (c 1.00, MeOH); ^{1}H-NMR (300 MHz, CD_3OD): δ 8.37 (1H, d, J = 7.5 Hz), 7.68 (2H, d, J = 8.0 Hz), 7.32 (2H, d, J = 8.0 Hz), 7.22 (H, d, J = 7.5 Hz), 6.15 (1H, t, J = 6.0 Hz), 5.14 (2H, s), 4.37-4.29 (1H, m), 4.00-3.92 (1H, m), 3.79 (1H, dd, J = 3.0, 12.0 Hz), 3.69 (1H, dd, J = 3.0, 12.0 Hz), 2.49-2.38 (1H, m), 2.17-2.06 (1H, m), 1.26 (12H, s); ^{13}C-NMR (75 MHz, $CDCl_3$): δ 163.7, 156.6, 156.5, 153.4, 144.9, 139.2, 134.9, 128.5, 128.4, 128.2, 127.2(3), 127.1(6), 95.6, 88.3, 87.5, 84.1, 70.5, 67.3, 61.4, 41.4, 24.2, 24.0, 17.4; FAB-LRMS m/z = 488 (MH^+); FAB-HRMS calcd for $C_{23}H_{31}N_3O_8B$= 488.2119, found 488.2216; MS (FAB) *m/z* 488 $[M+H]^+$; HRMS (FAB): Calcd for $C_{23}H_{31}N_3O_8B$ $[M+H]^+$: 488.2119. Found: 488.2119.

1-10. 5'-*O*-(4,4'-Dimethoxytrityl)-(4-(4,4,5,5-tetramethyl-1,3,2-dioxaborolan-2-yl)benzyl)-2'-deoxy thymidine (21)

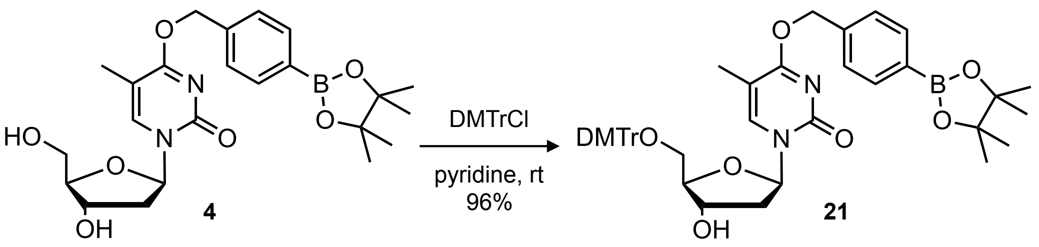

Compound **4** (450 mg, 0.945 mmol) was dissolved in dry pyridine (10 mL) and DMTrCl (384 mg, 1.13 mmol) was addedat room temperature. The reaction mixture was stirred for 3 h at room temperature and quenched by addition of MeOH at 0 °C with 10 min. stirring. After the solvent was removed in vacuo, the residue was purified by a silica gel column chromatography, eluted with $CHCl_3$/MeOH (19:1 with 0.5% Et_3N) to give compound **21** (705 mg, 96%) as a white foam. IR (KBr): *v* 3455 (-OH), 2978 (Ar C-H), 1700 (C=O), 1642 (C=N) cm^{-1} $[\alpha]_D^{24}$ 8.8 (c 1.00, $CHCl_3$); ^1H-NMR (300 MHz, $CDCl_3$): δ 8.54-8.50 (2H, m), 7.79-7.74 (2H, m), 7.69-7.63 (2H, m), 7.51-7.38 (4H, m), 7.32-7.18 (6H, m), 6.85-6.78 (4H, m), 6.50-6.42 (1H, m), 5.11(2H, s), 4.63-4.57 (1H, m), 4.12-4.07 (1H, m), 3.74 (6H, s), 3.51-3.45 (1H, m), 3.38-3.31 (1H, m), 2.48-2.24 (2H, m), 1.55 (3H, s), 1.31 (12H, s); ^{13}C-NMR (75 MHz, $CDCl_3$): δ 163.3, 163.2, 158.4, 150.7, 150.6, 149.1, 144.2, 139.8, 136.7, 136.2, 135.2, 135.1, 134.7, 133.8, 129.9, 128.9, 128.2, 128.1, 127.9, 127.7, 127.3, 126.9, 123.8, 113.0, 110.1, 110.0, 86.6, 86.1, 85.3, 83.5, 77.2, 74.7, 71.4, 63.3, 55.0, 44.3, 41.0, 24.6, 12.4; MS (FAB) *m/z* 783 $[M+Na]^+$; HRMS (FAB): Calcd for $C_{44}H_{49}N_2O_9BNa$ $[M+Na]^+$: 783.3429. Found: 783.3436.

1-11. 3-*O*-{2-Cyanoethyl(diisopropylamino)phosphino}-5'-*O*-(4,4'-dimethoxytrityl)- (4-(4,4,5,5-tetramethyl-1,3,2-dioxaborolan-2-yl)benzyl)-2'-deoxy thymidine (11)

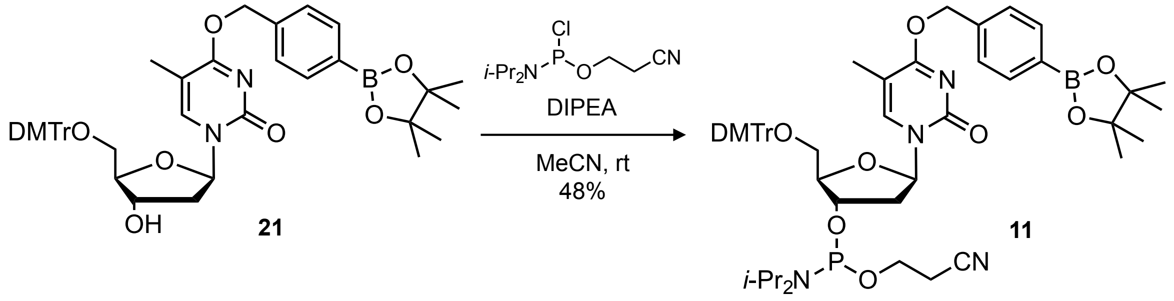

Compound **21** (663 mg, 0.850 mmol) was dissolved in dry DCM (10 mL) and *N*,*N*-diisopropylamine (440 µL, 2.55 mmol) and 2-cyanoethyl-*N*,*N'*-diisopropylchlorophosphoramidite (230 µL, 1.02 mmol) were added at room temperature. The reaction mixture was stirred for 2 h and partitioned between AcOEt and H_2O. The separated organic layer was washed with brine, dried over Na_2SO_4 and concentrated in vacuo. The residue was purified by a silica gel column chromatography, eluted with hexane/AcOEt (6:4) to give compound **11** (400 mg, 48%) as a white foam. IR (KBr): *v* 2244 (C≡N), 1671 (C=N) cm^{-1} ^1H-NMR (300 MHz, $CDCl_3$): δ 8.03-7.93 (1H, m), 7.83 (2H, d, *J*= 7.5 Hz), 7.48-7.39 (4H, m), 7.35-7.24 (7H, m), 6.89-6.79 (4H, m), 6.49-6.36 (1H, m), 5.43 (2H, s), 4.78-4.59 (1H, m), 4.30-4.17 (1H, m), 3.77 (3H, s), 3.76 (3H, s), 3.66-3.45 (4H, m), 3.45-3.27 (2H, m), 2.79-2.26 (4H, m), 1.49 (3H, s), 1.34 (12H, s), 1.26-1.03 (12H, m); ^{13}C-NMR (75 MHz, $CDCl_3$): δ 169.8, 169.7, 158.3, 155.5, 155.4, 144.0, 139.6, 138.6, 135.0, 134.9, 134.7, 129.9, 129.8, 127.9, 127.8, 127.6, 126.8, 117.4, 117.2, 112.9, 104.6, 104.5, 86.5, 86.4, 85.9, 83.5, 77.2, 74.5, 68.2,

58.0, 57.8, 54.9, 54.8, 42.9, 42.8 (d, *J* (C, P) = 5.0 Hz)), 42.7, 24.5, 24.4, 24.3, 24.2, 24.1 (d, *J* (C, P) = 7.0 Hz), 20.1, 19.9, 19.8, 19.7, 11.3, 44.9 (d, *J* (C, P) = 5.0 Hz), 24.4, 24.3, 22.8, 22.7, 20.2; ^{31}P-NMR (120 MHz, $CDCl_3$): δ 149.57, 148.96; FAB-LRMS m/z = 961 (MH^+); FAB-HRMS calcd for $C_{53}H_{67}BN_4O_{10}P$= 961.4688, found 961.4697.

2. ^{1}H-, ^{13}C- and ^{31}P-NMR spectra of new compounds

2-1. ^{1}H spectrum of compound 17

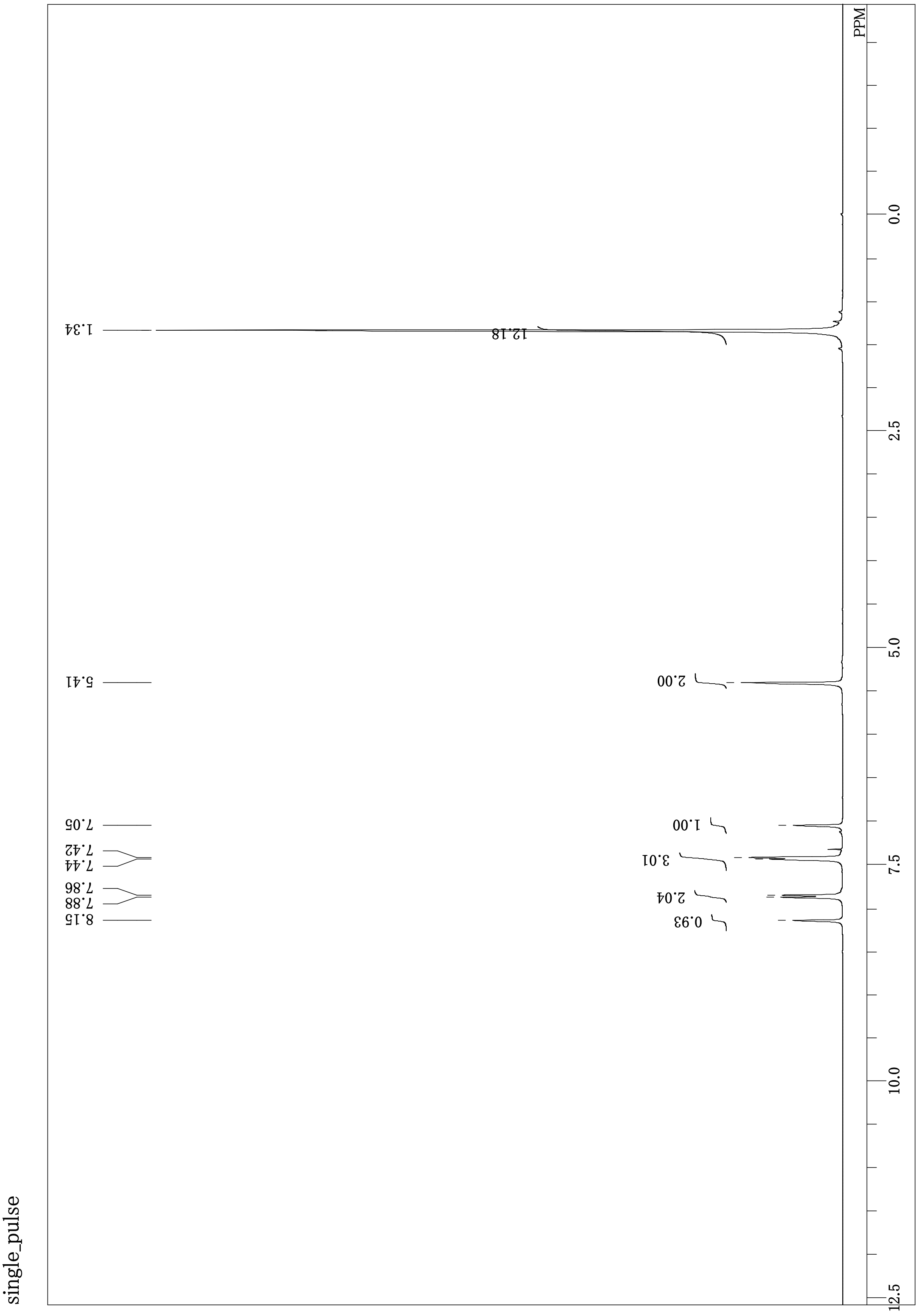

2-2. ^{13}C spectrum of compound 17

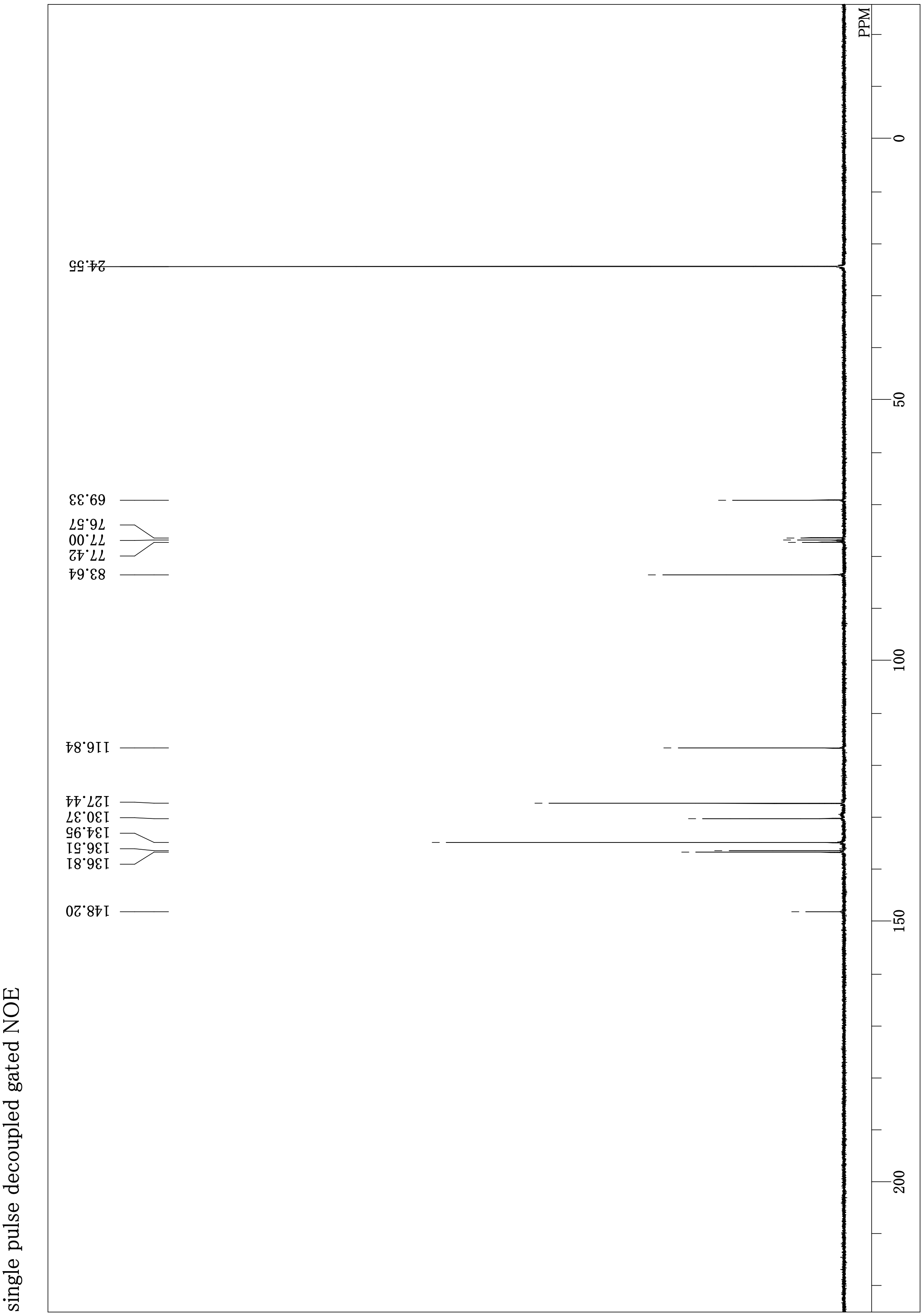

2-3. ^{1}H spectrum of compound 18

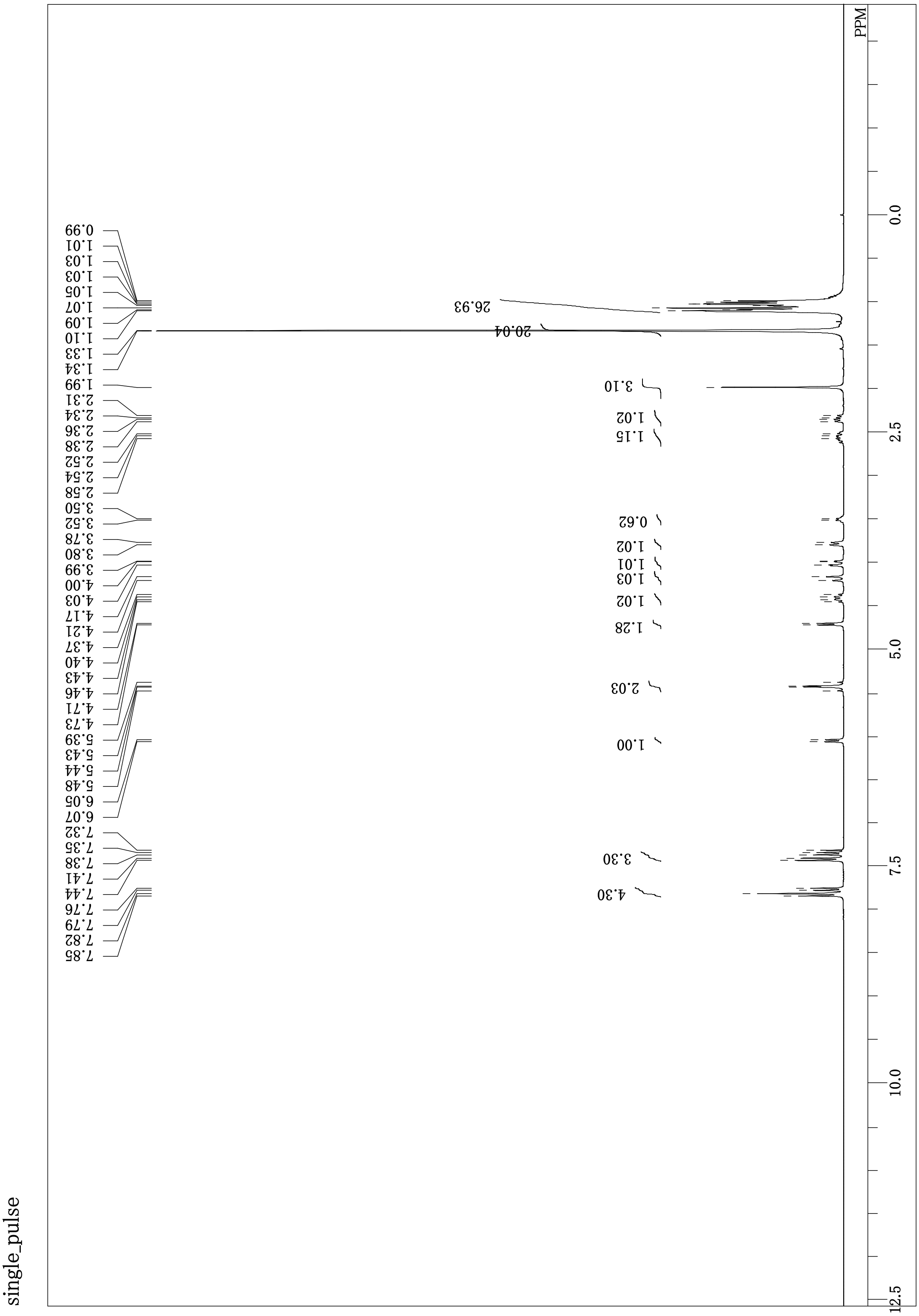

2-4. ^{13}C spectrum of compound 18

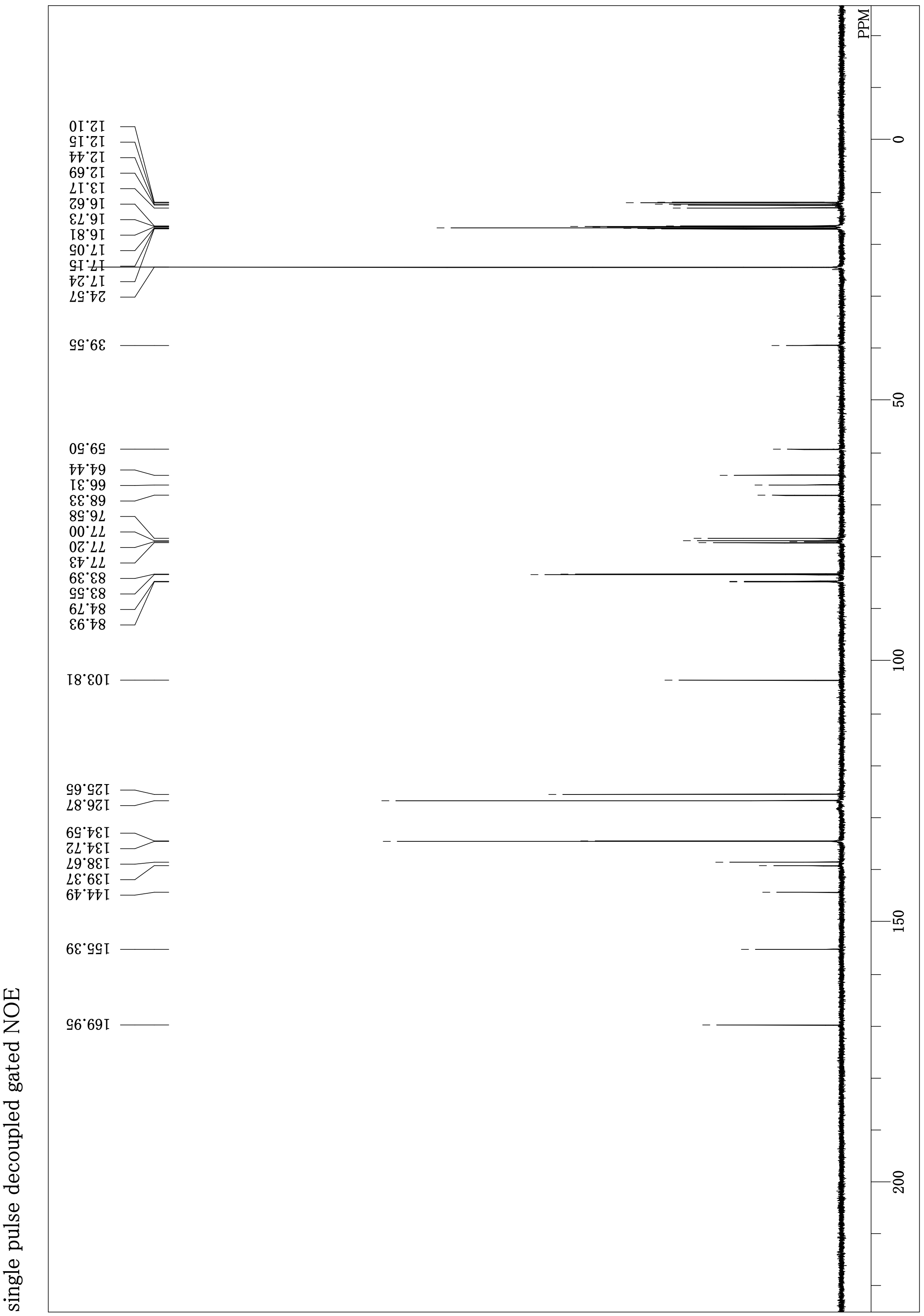

2-5. ^{1}H spectrum of compound 4

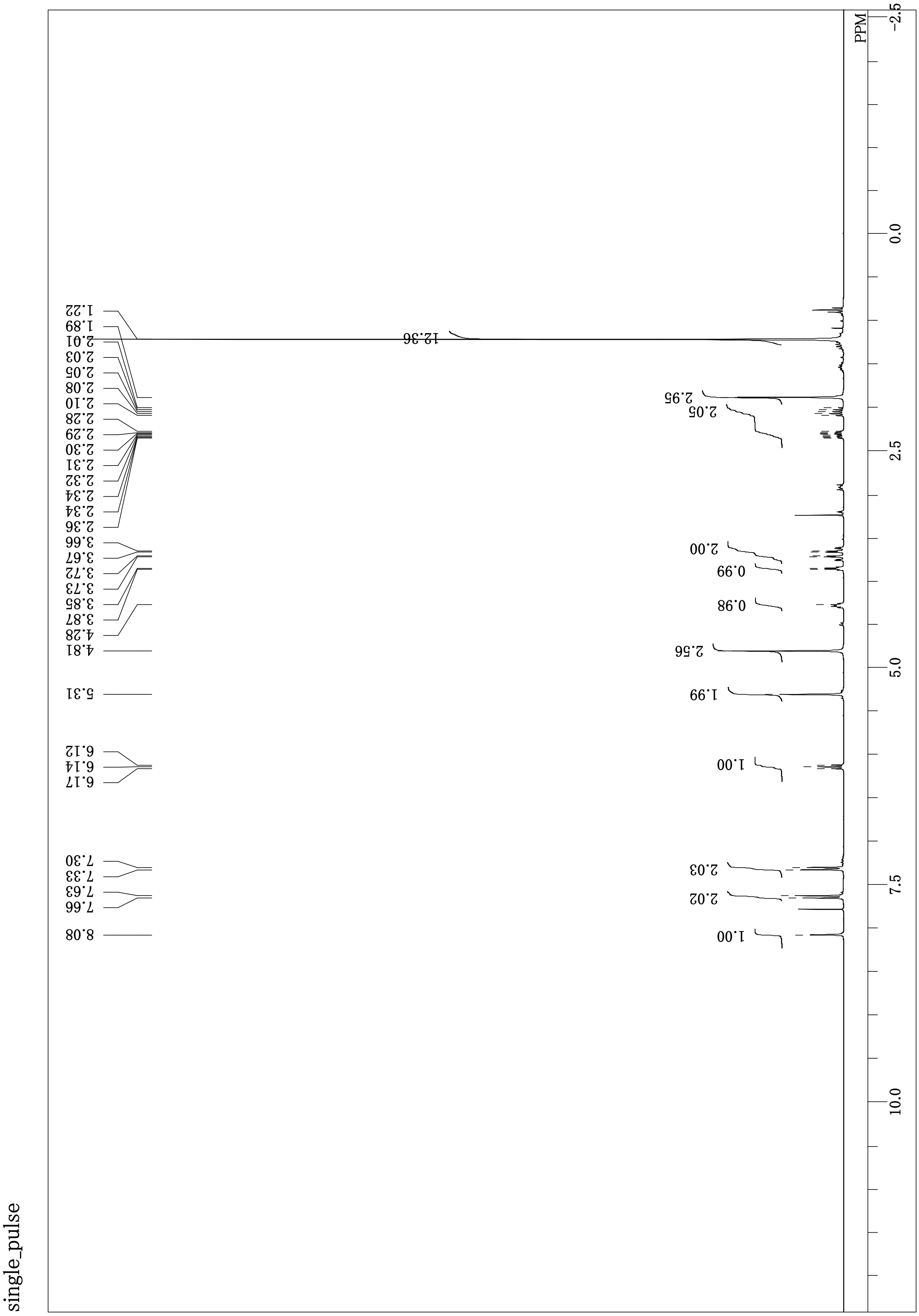

2-6. ^{13}C spectrum of compound 4

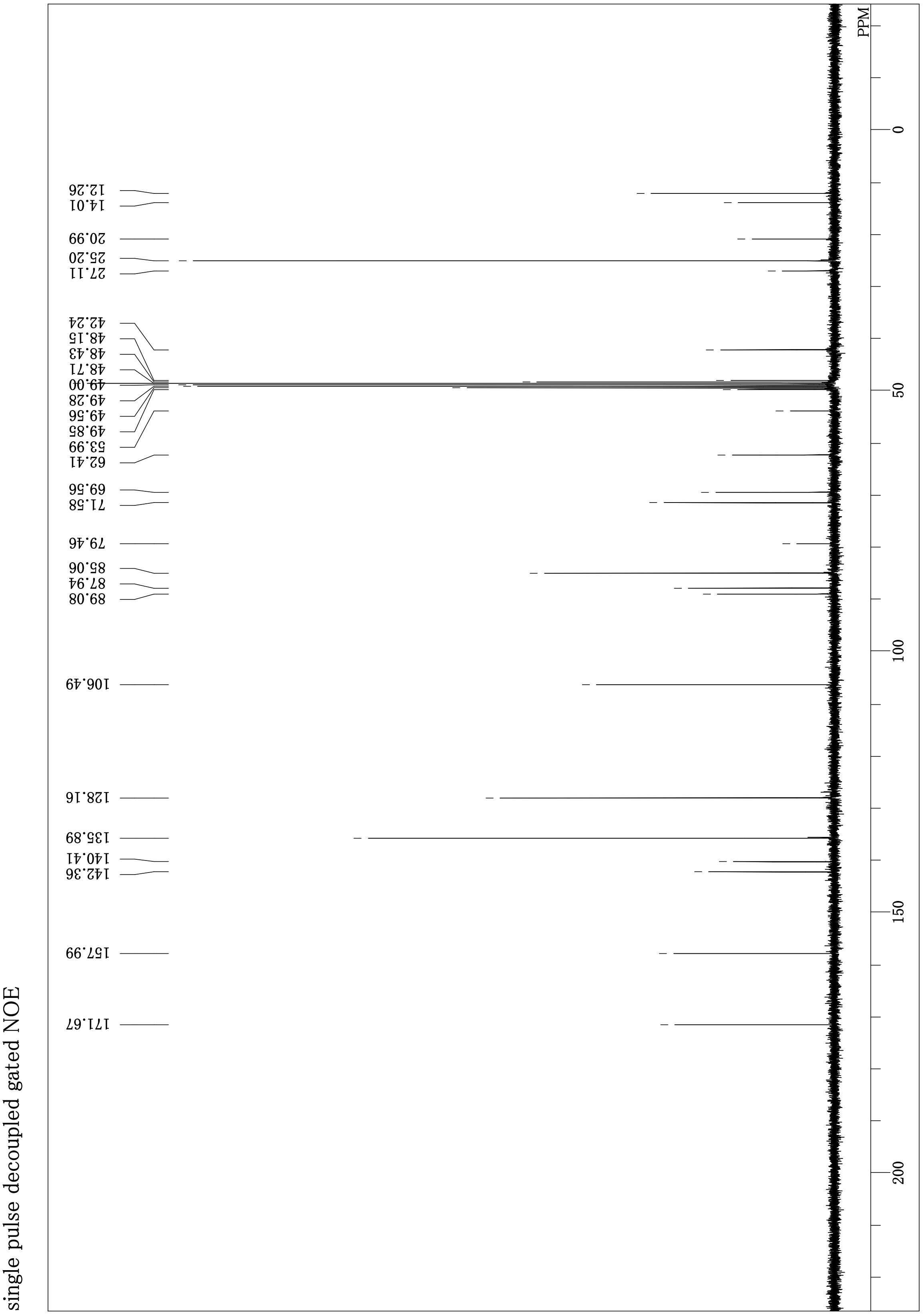

2-7. ^{1}H spectrum of compound 6

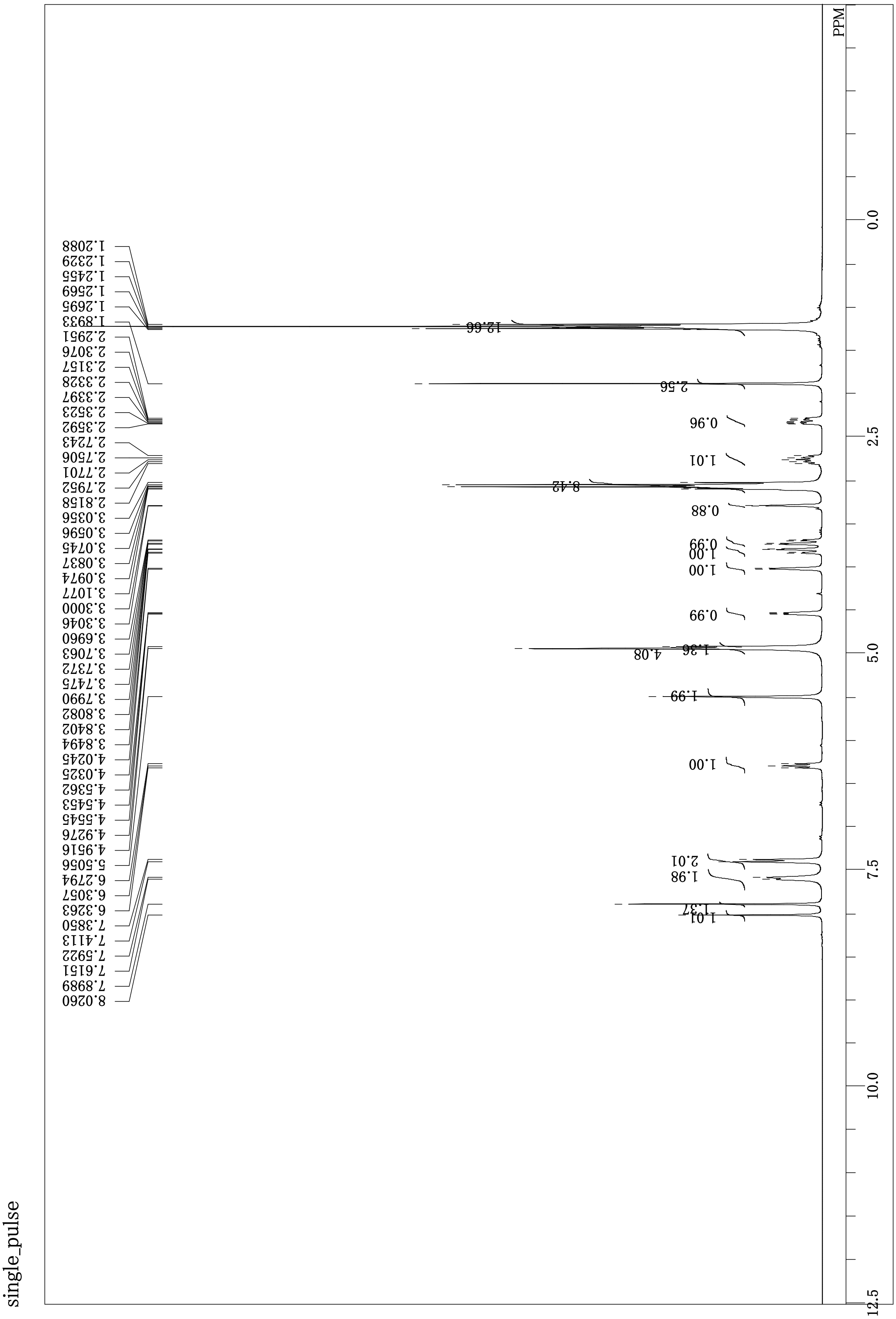

2-8. ^{13}C spectrum of compound 6

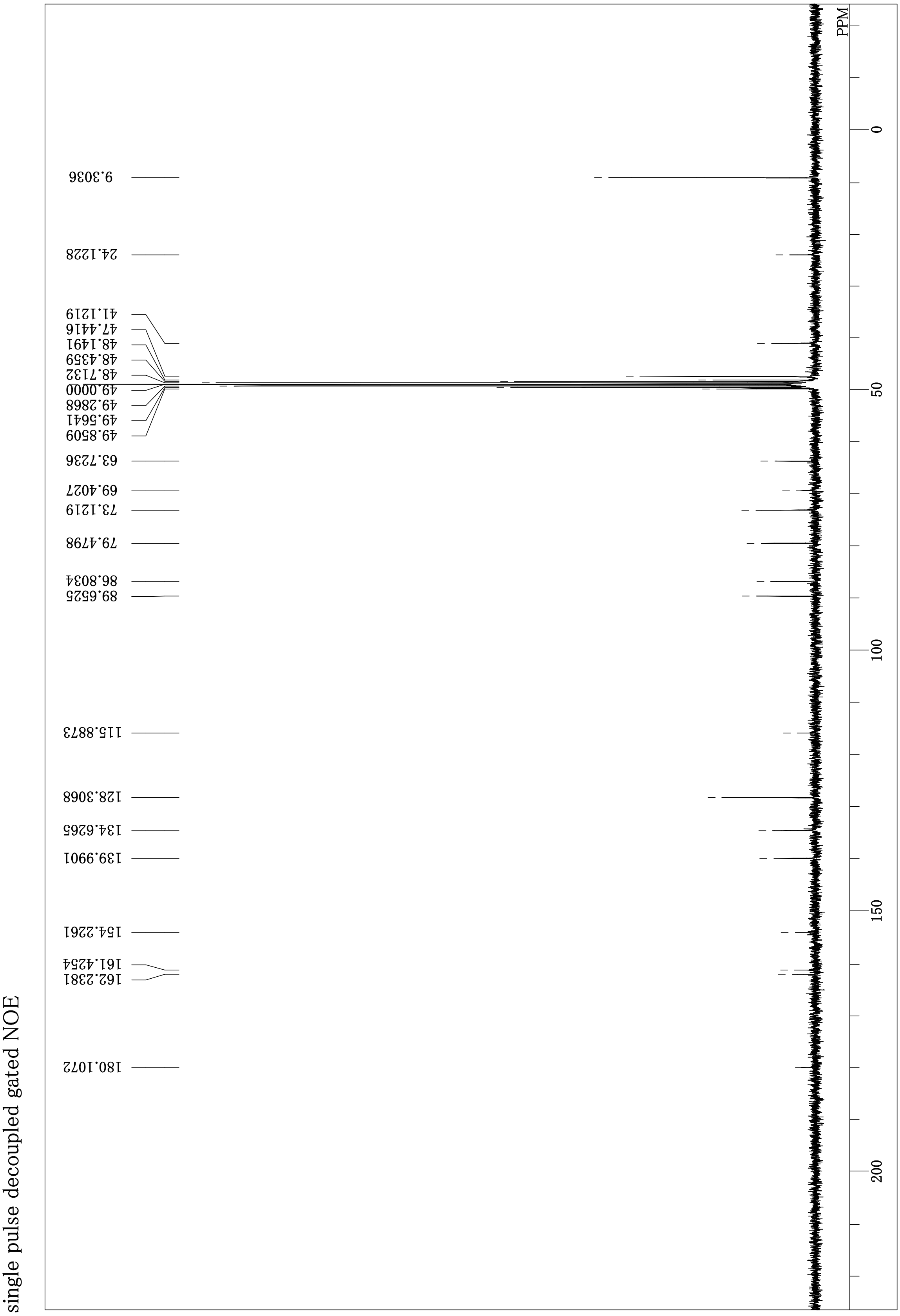

2-9. ^{1}H spectrum of compound 19

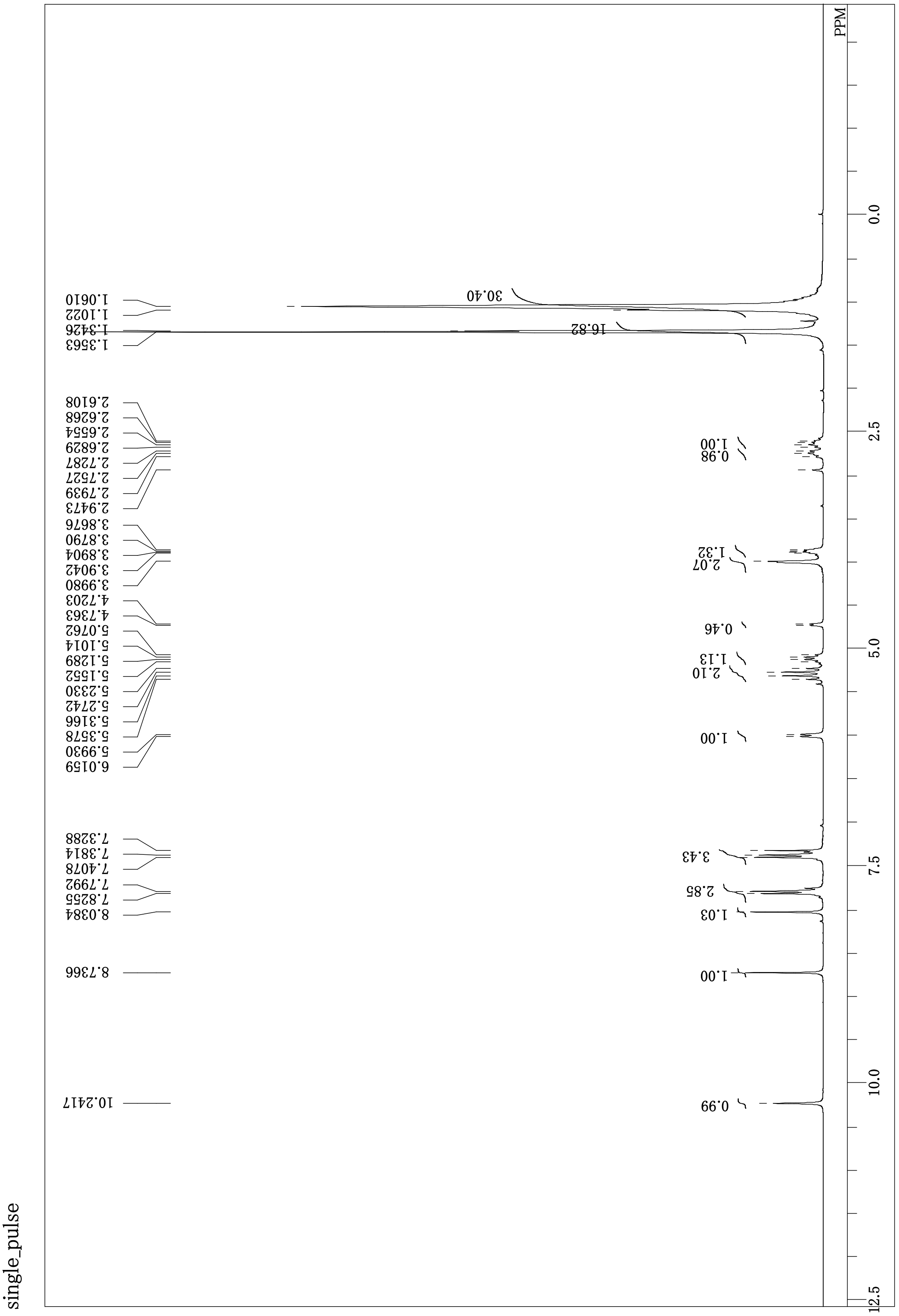

2-10. ^{13}C spectrum of compound 19

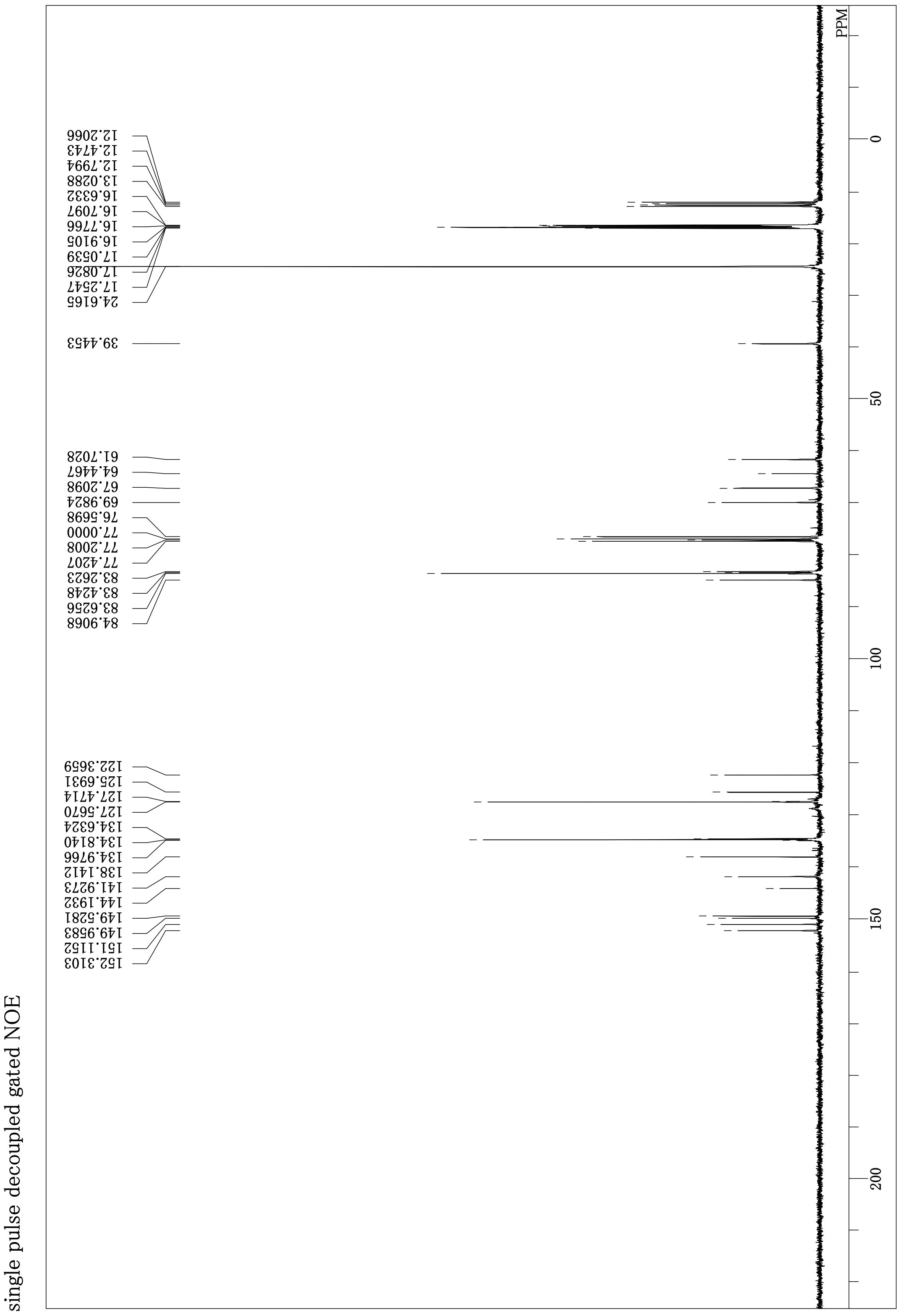

2-11. ^{1}H spectrum of compound 8

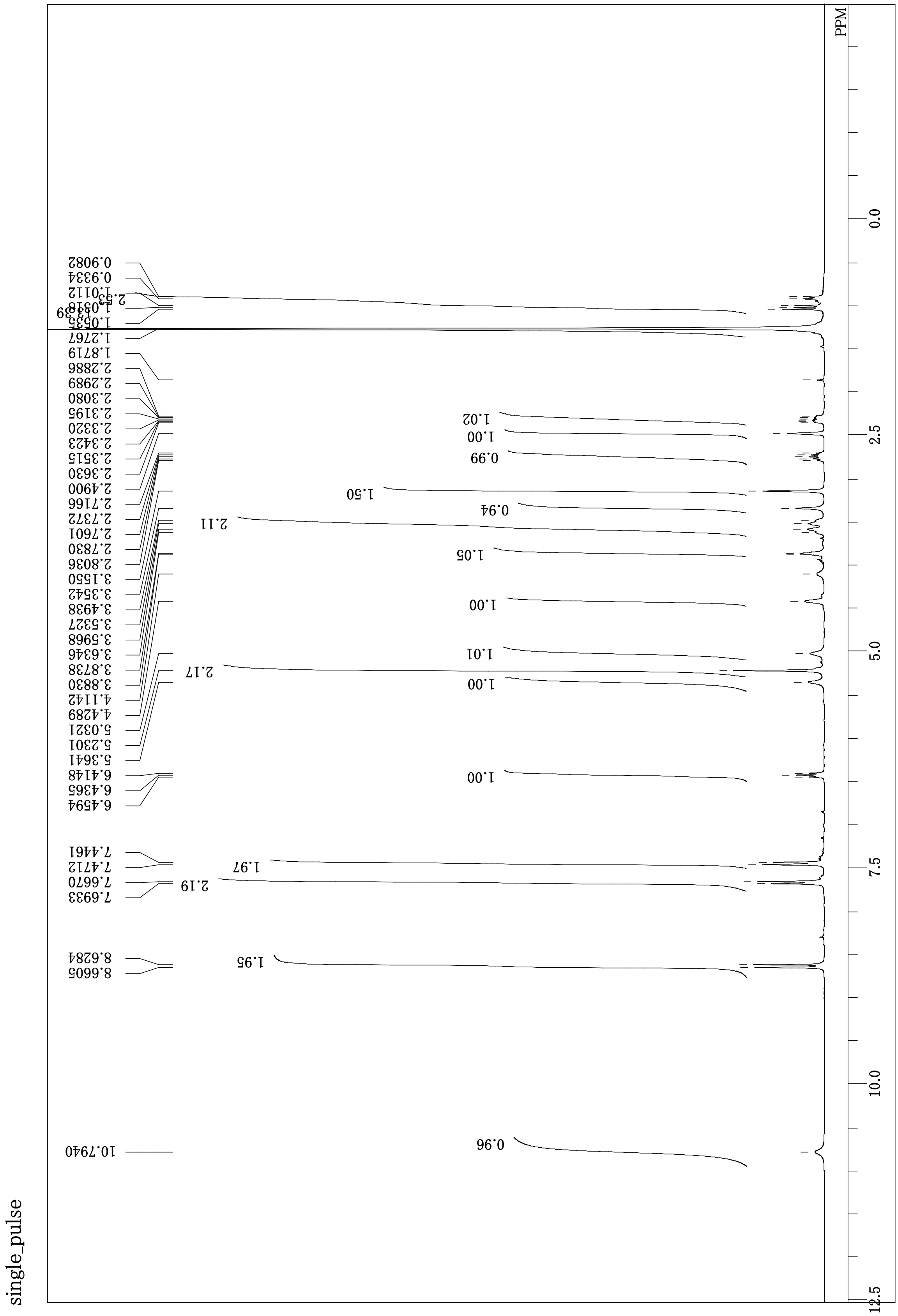

2-12. ^{13}C spectrum of compound 8

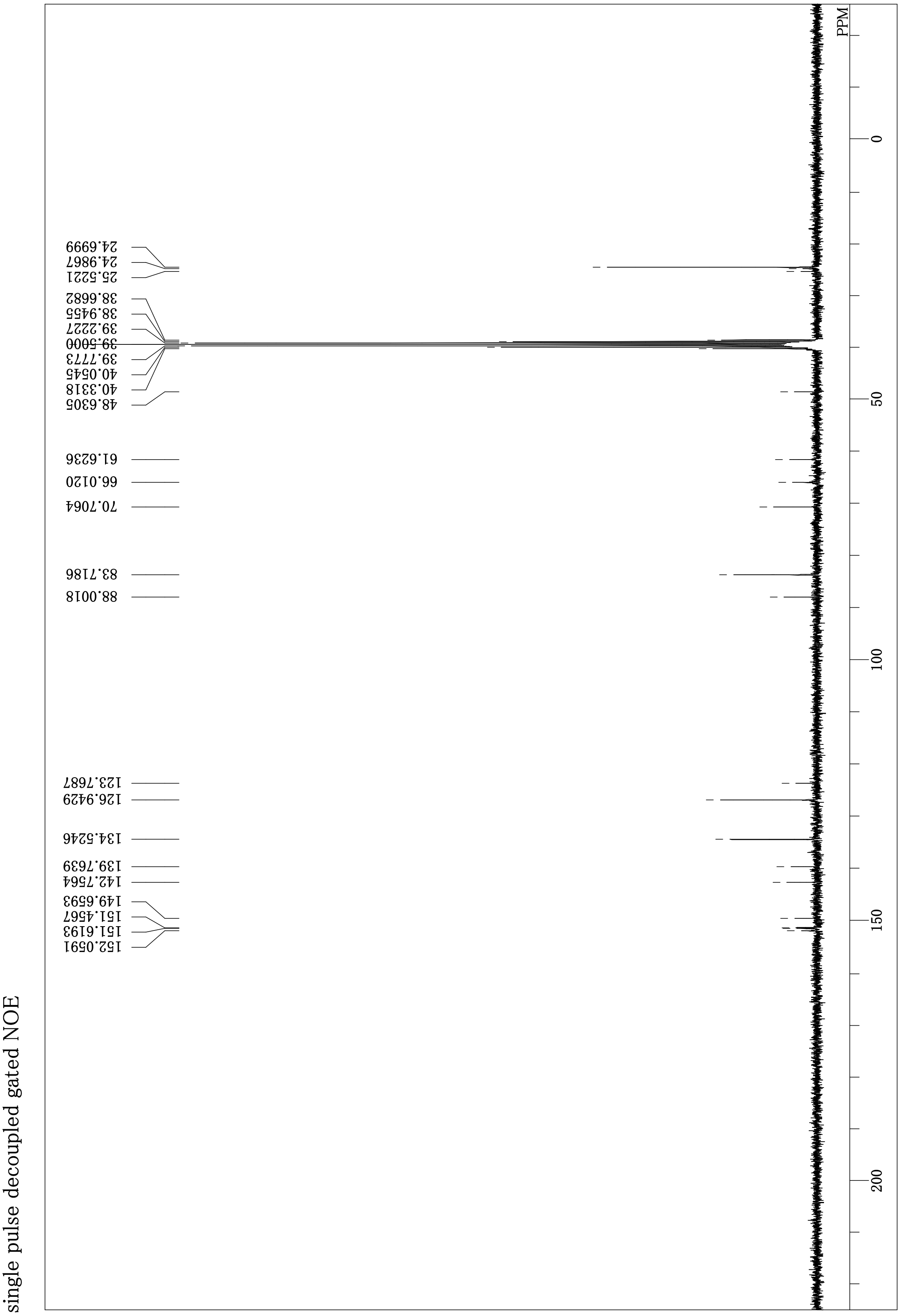

2-13. ^{1}H spectrum of compound 20

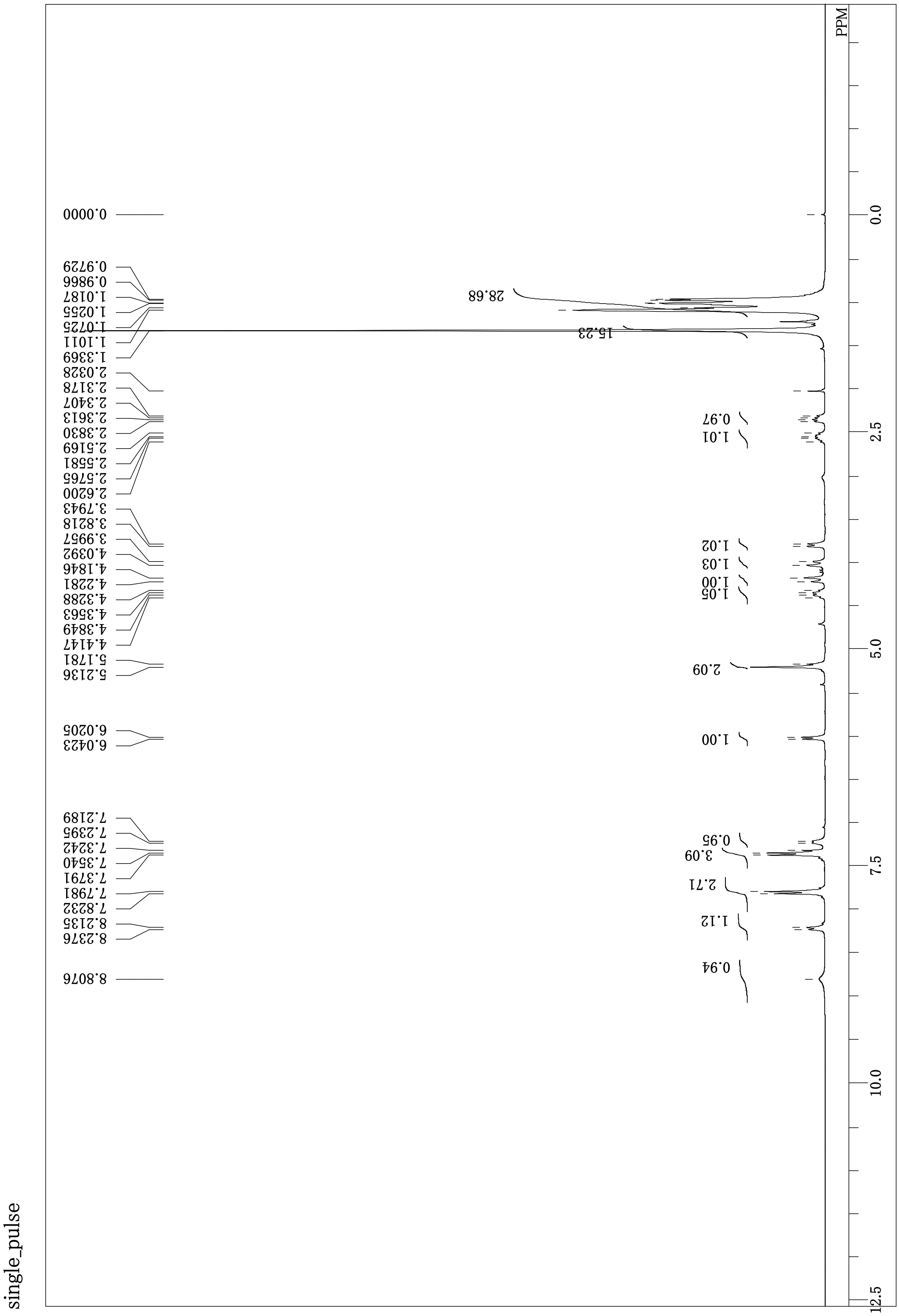

2-14. ^{13}C spectrum of compound 20

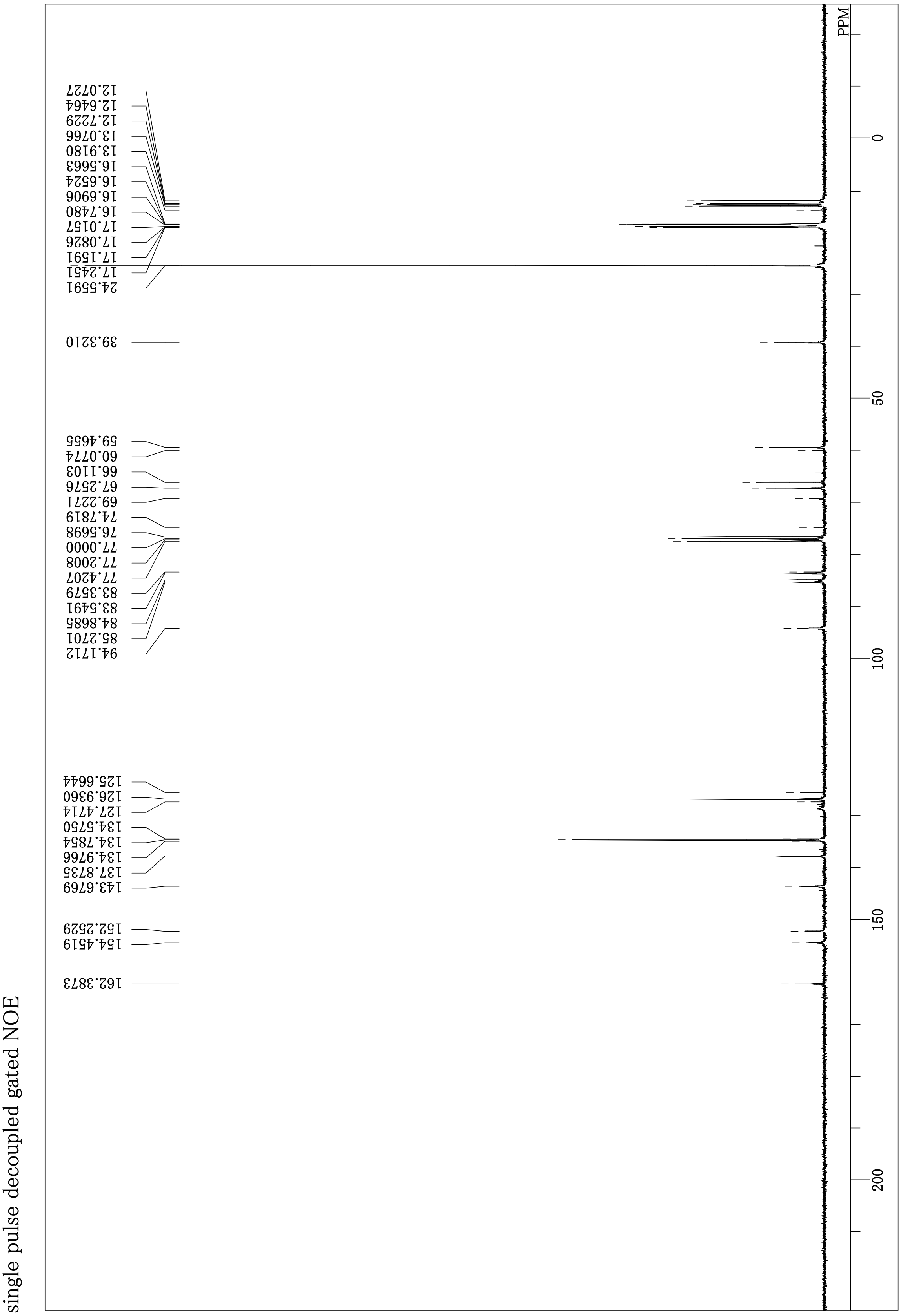

2-15. ^{1}H spectrum of compound 10

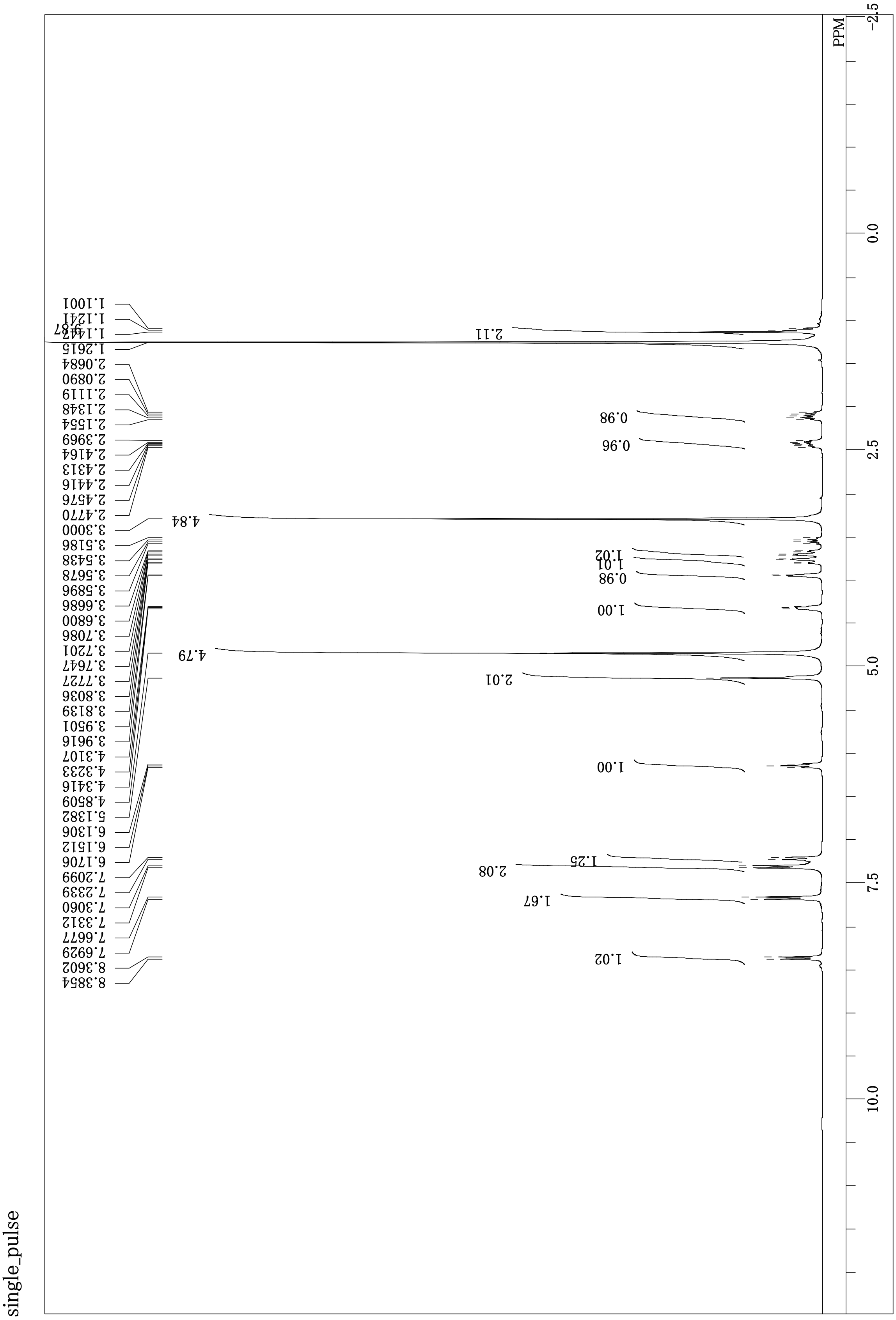

2-16. ^{13}C spectrum of compound 10

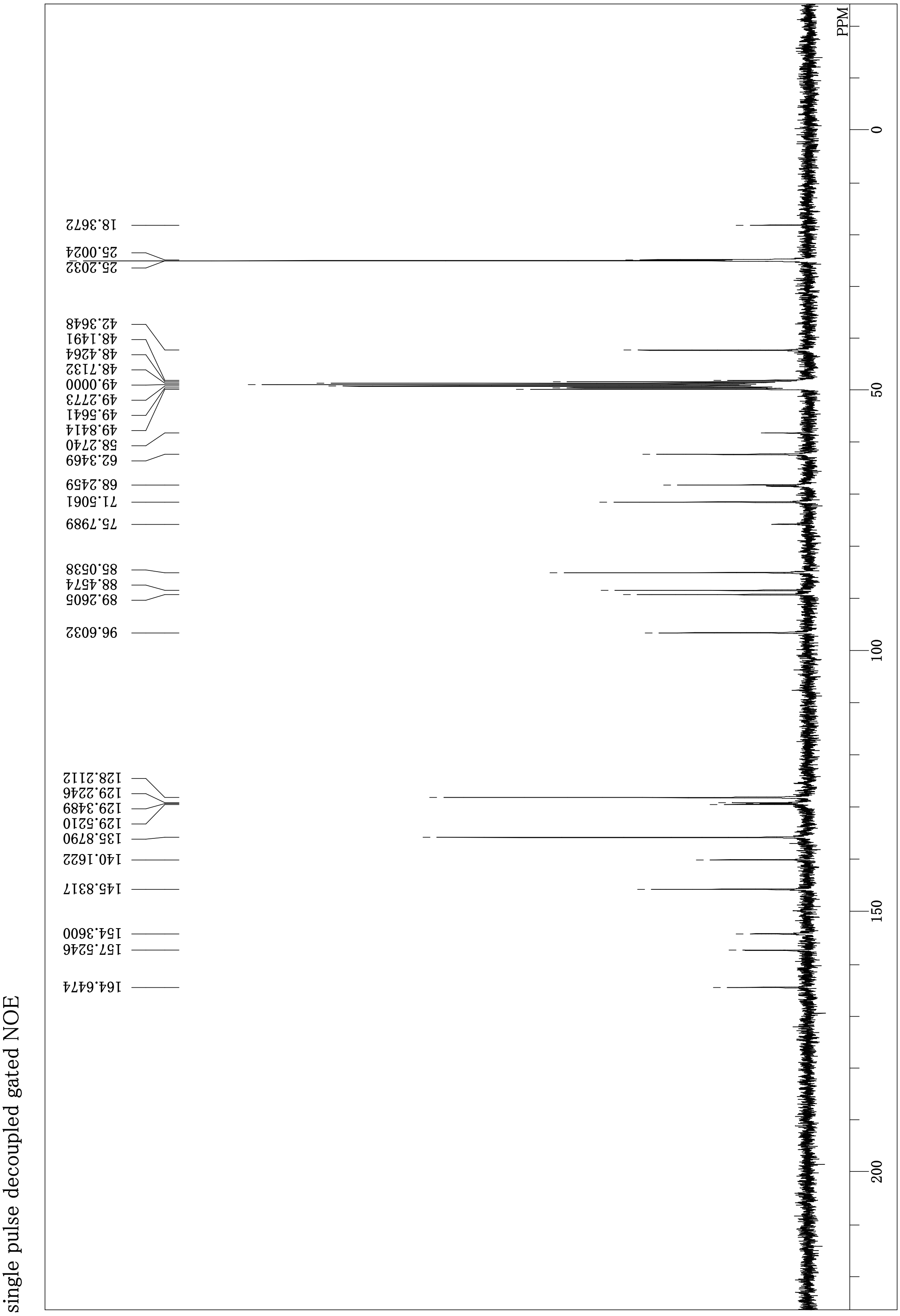

2-17. ^{1}H spectrum of compound 21

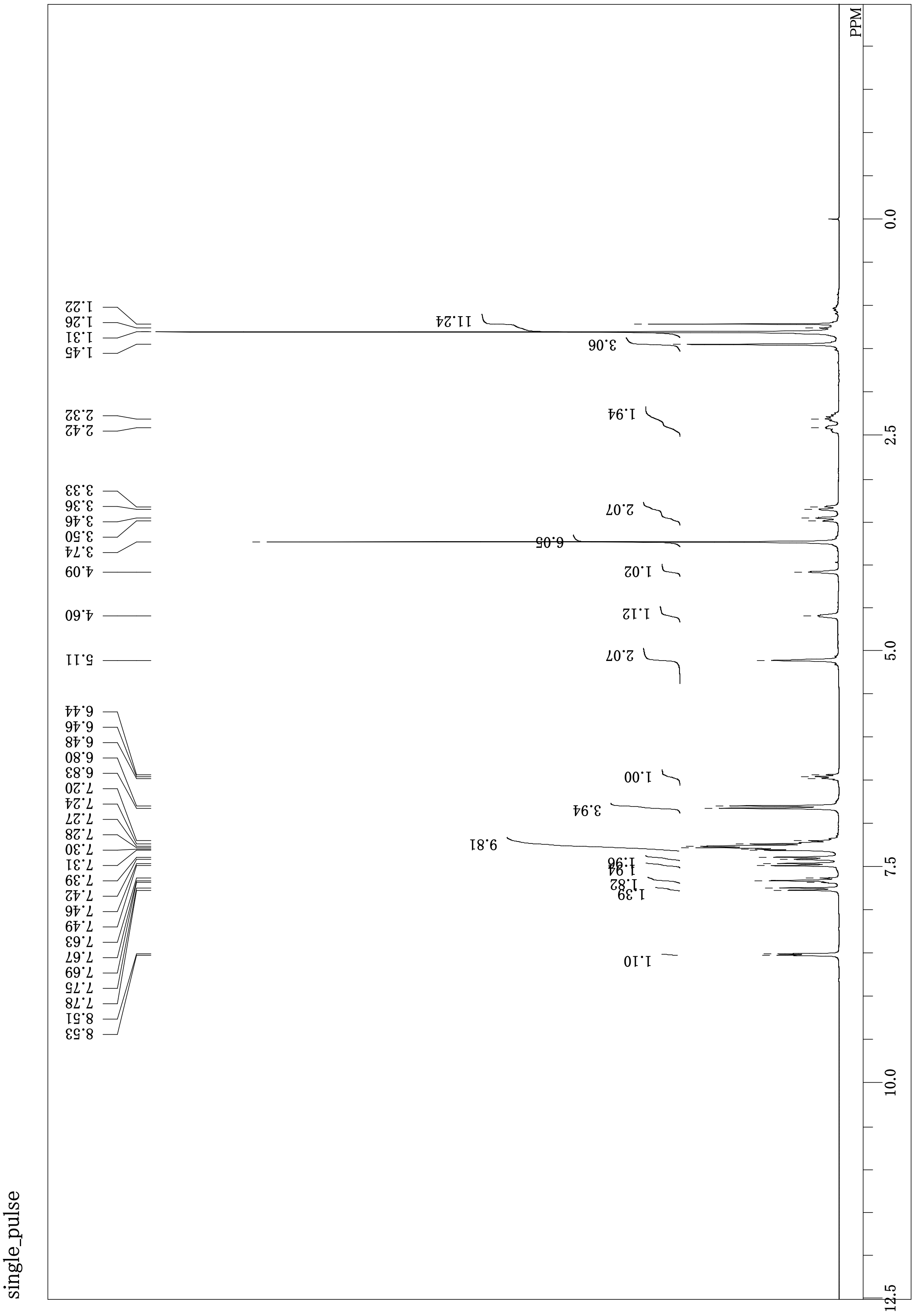

2-18. ^{13}C spectrum of compound 21

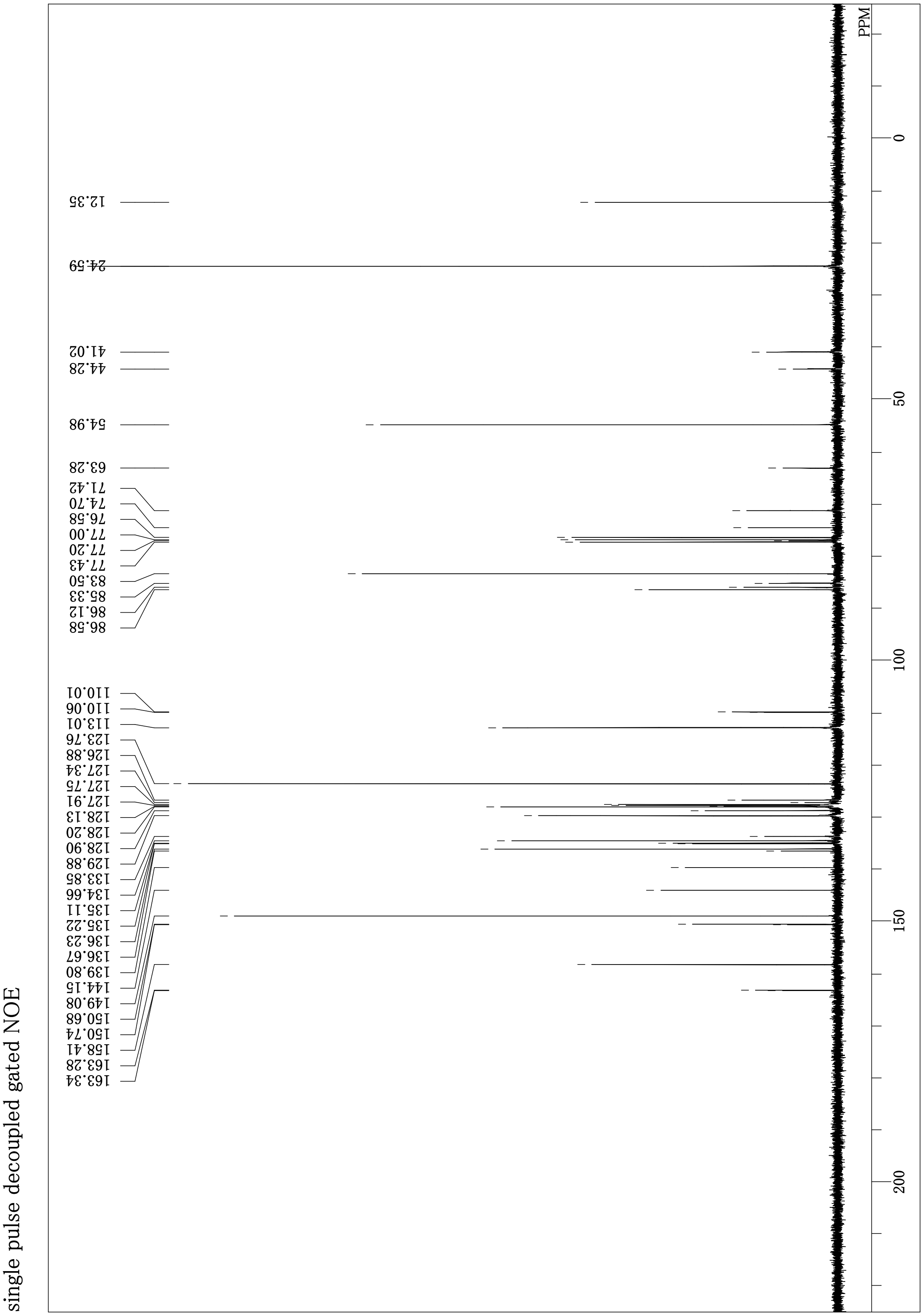

2-19. ^{1}H spectrum of compound 11

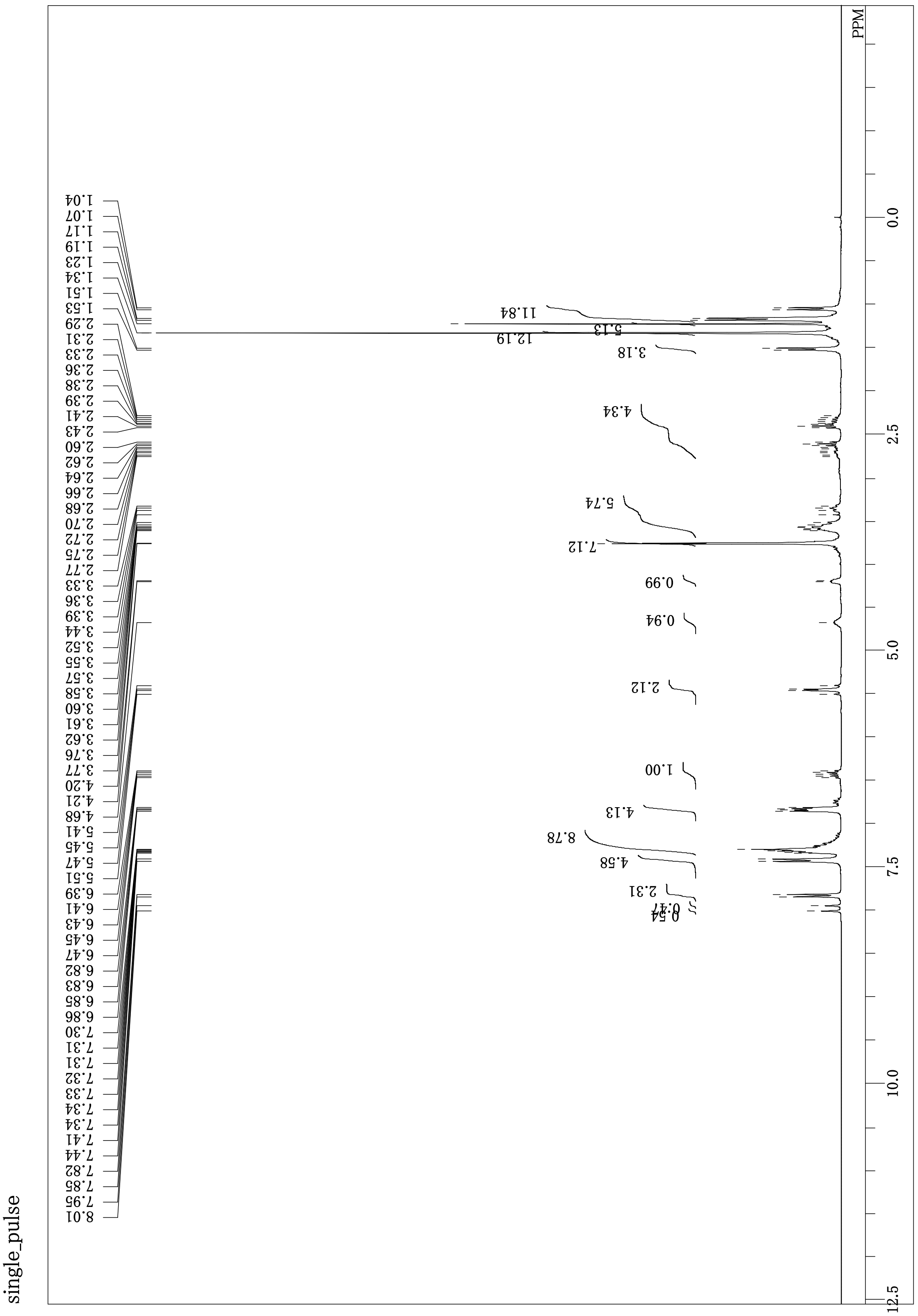

2-20. ^{13}C spectrum of compound 11

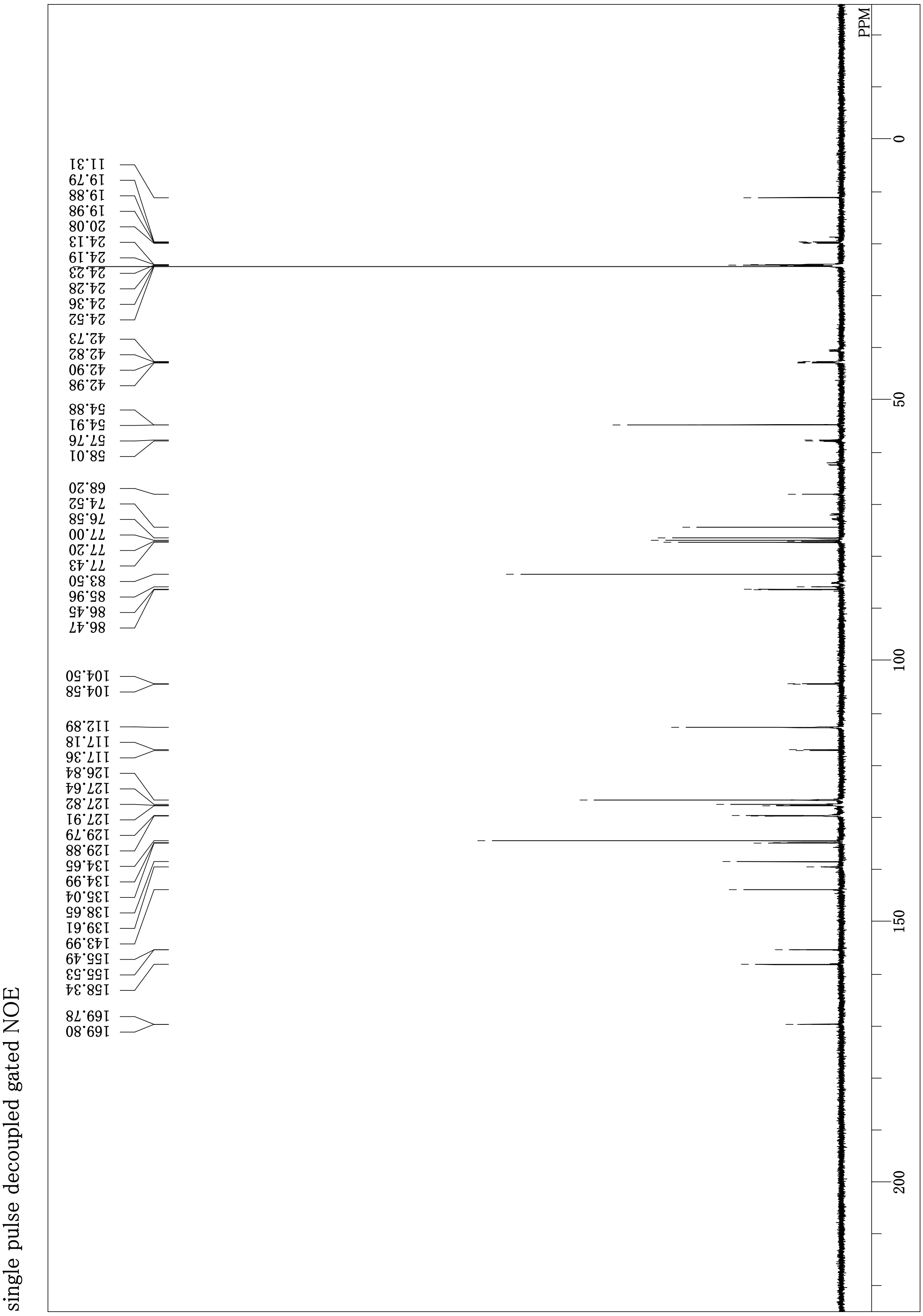

2-21. ^{31}P spectrum of compound 11

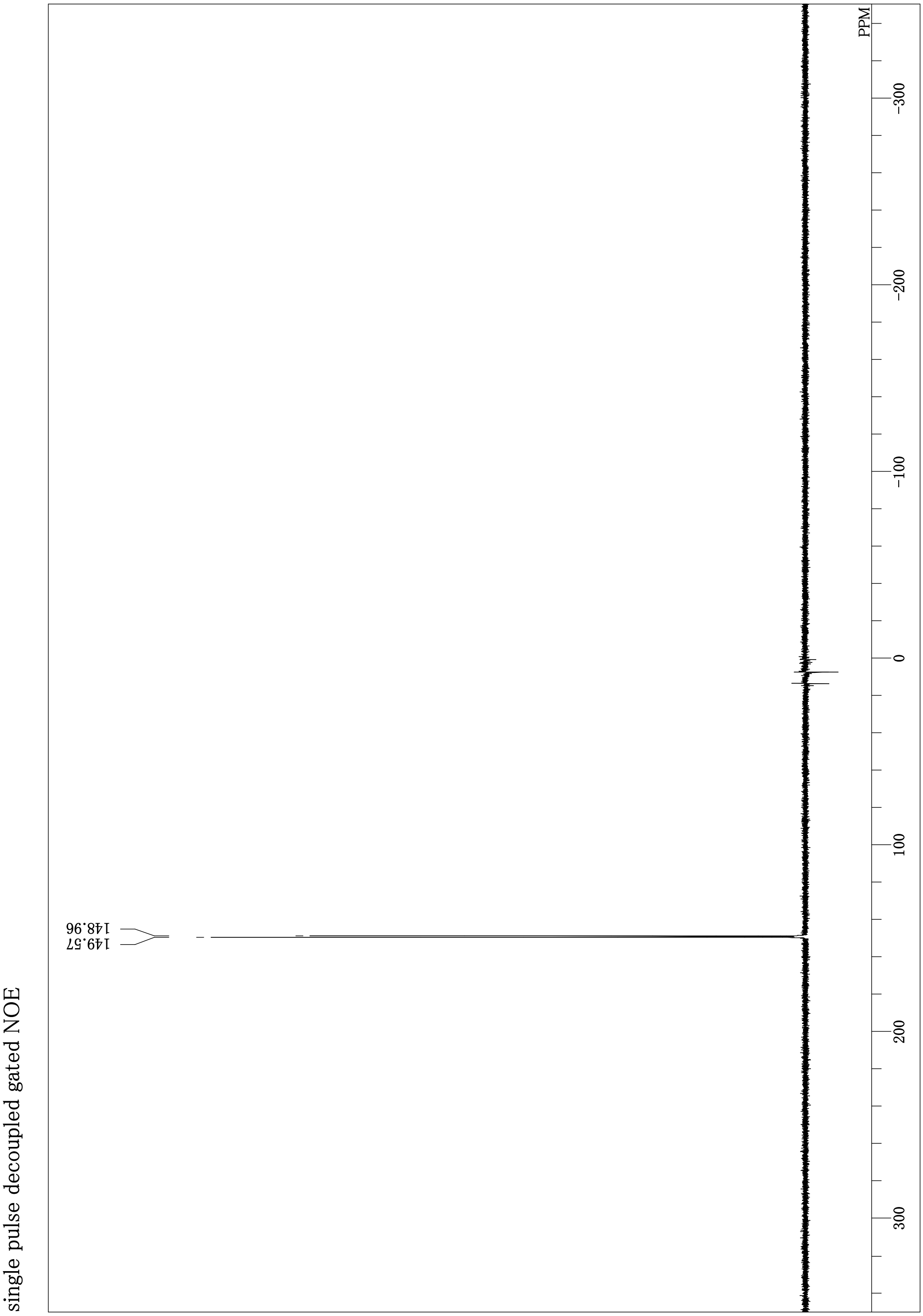

3. H_2O_2-decaging of boronated nucleosides

3-1. HPLC chromatograms of **dA**[Bpin] after H_2O_2 addition at different time points.

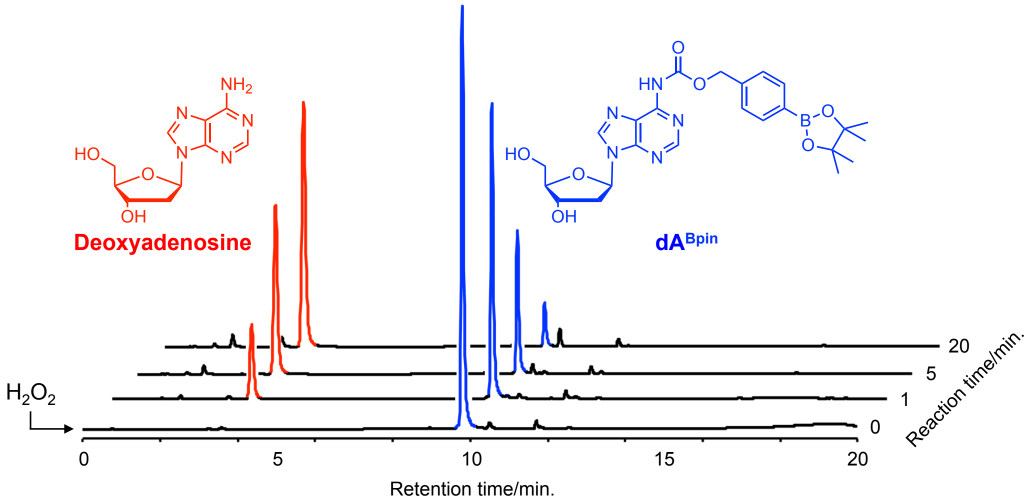

3-2. HPLC chromatograms of **dC**[Bpin] after H_2O_2 addition at different time points.

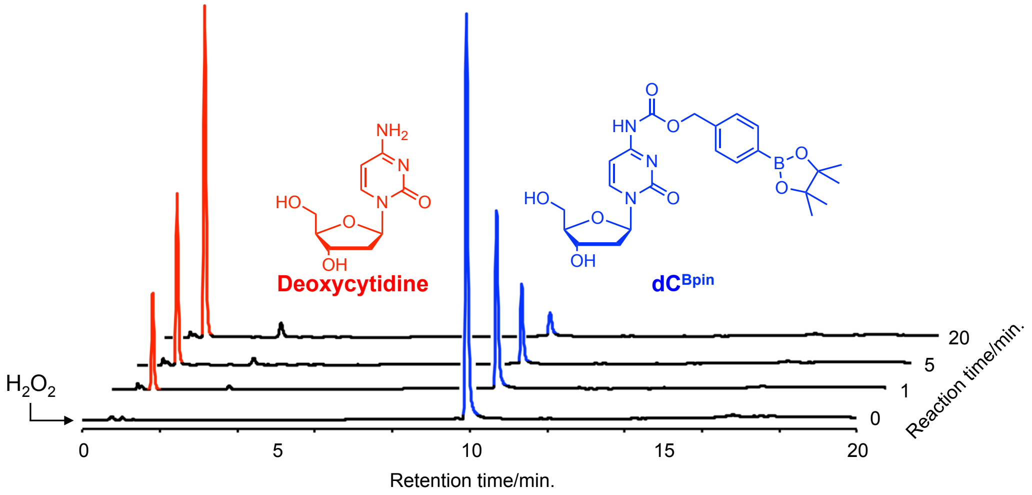

3-3. HPLC chromatograms of **dG**[Bpin] after H_2O_2 addition at different time points.

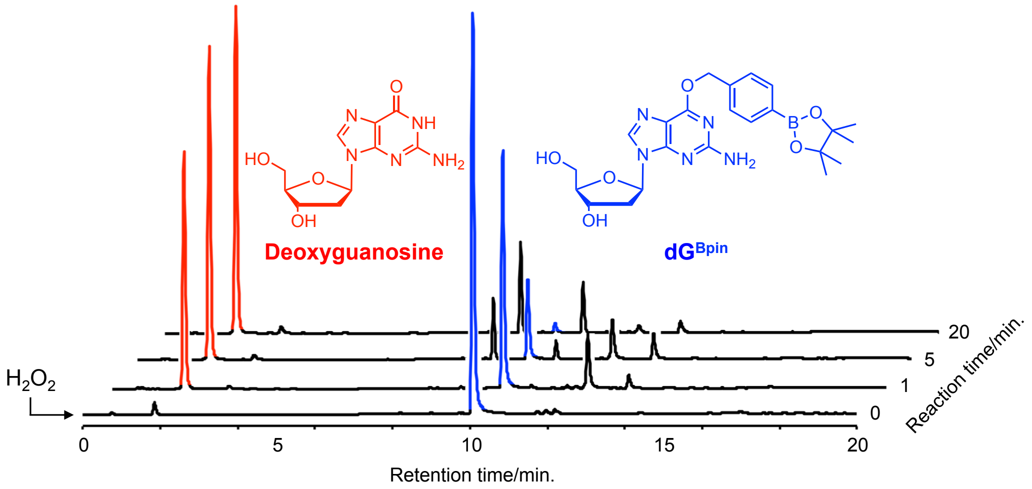

4. Peroxynitrite ($ONOO^-$)-decaging of dT^{Bpin}

4-1. HPLC chromatograms of **dT^{Bpin}** after peroxynitrite ($ONOO^-$) addition at different time points.

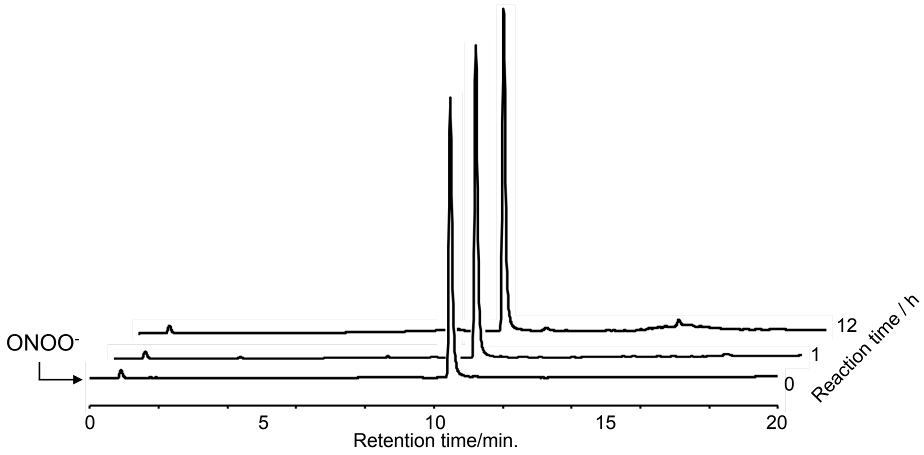

4. ESI and MALDI-TOF MS analysis of dT^B-modified ODNs

4-1. ON 14 5'-d(GCGTTT^{B}TTTCGT)-3'

HPLC

Column: Waters XBridge™ OST C18 2.5 µm, 4.6 x 50 mm

Gradient: 8-16%MeCN (over 30 min) in triethylammonium acetate butter (pH 7.0, 0.1 M)

Flow rate: 1.0 mL/min

Column temperature: 50 °C

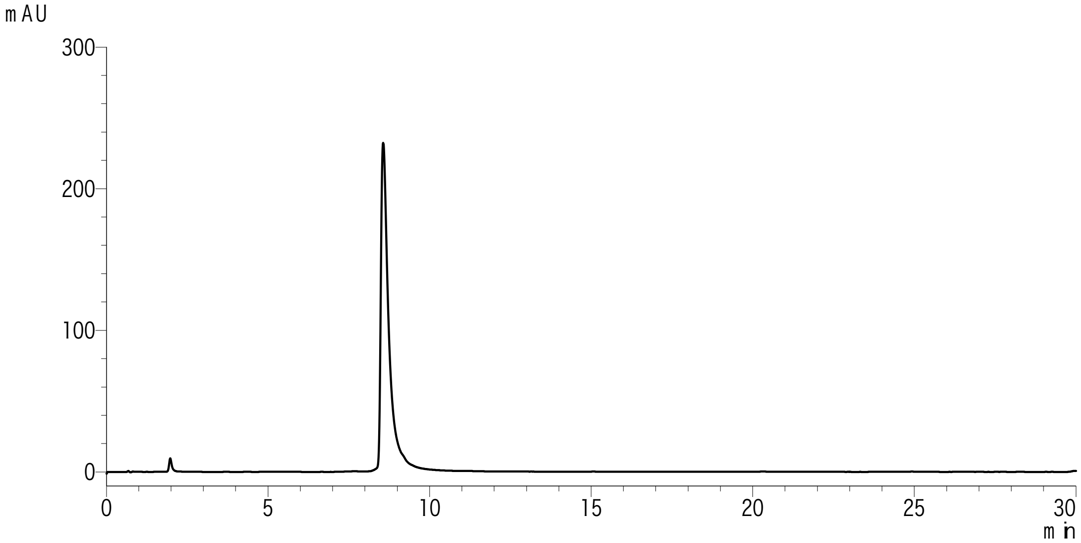

MALDI-TOF MS

Calcd. 3766.30 [M-H]$^-$ / citric acid adduct 3922.39 [M-H]$^-$

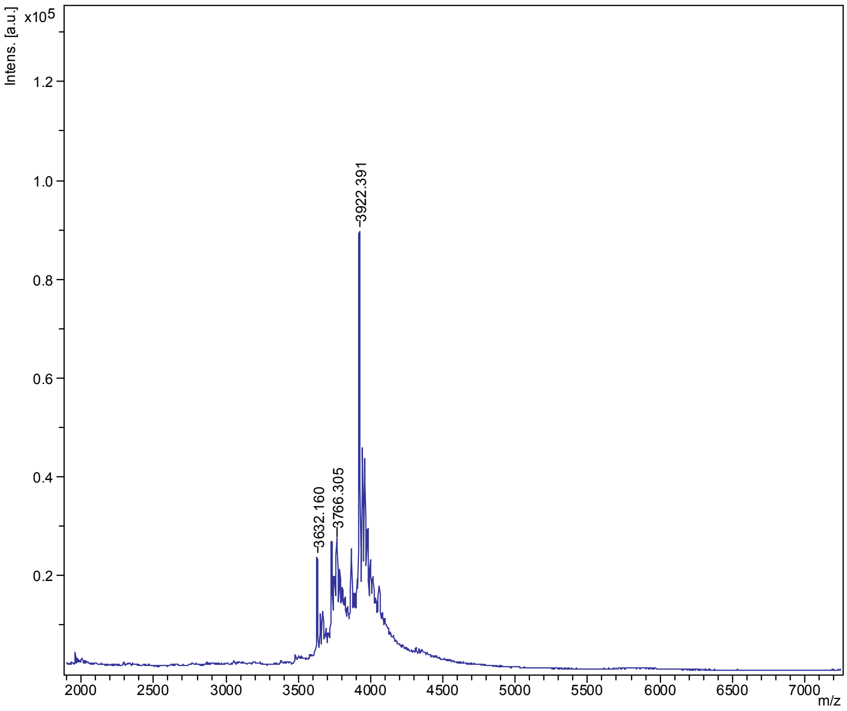

ESI MS

Calcd. 3731.30 [M - 2 x H_2O]

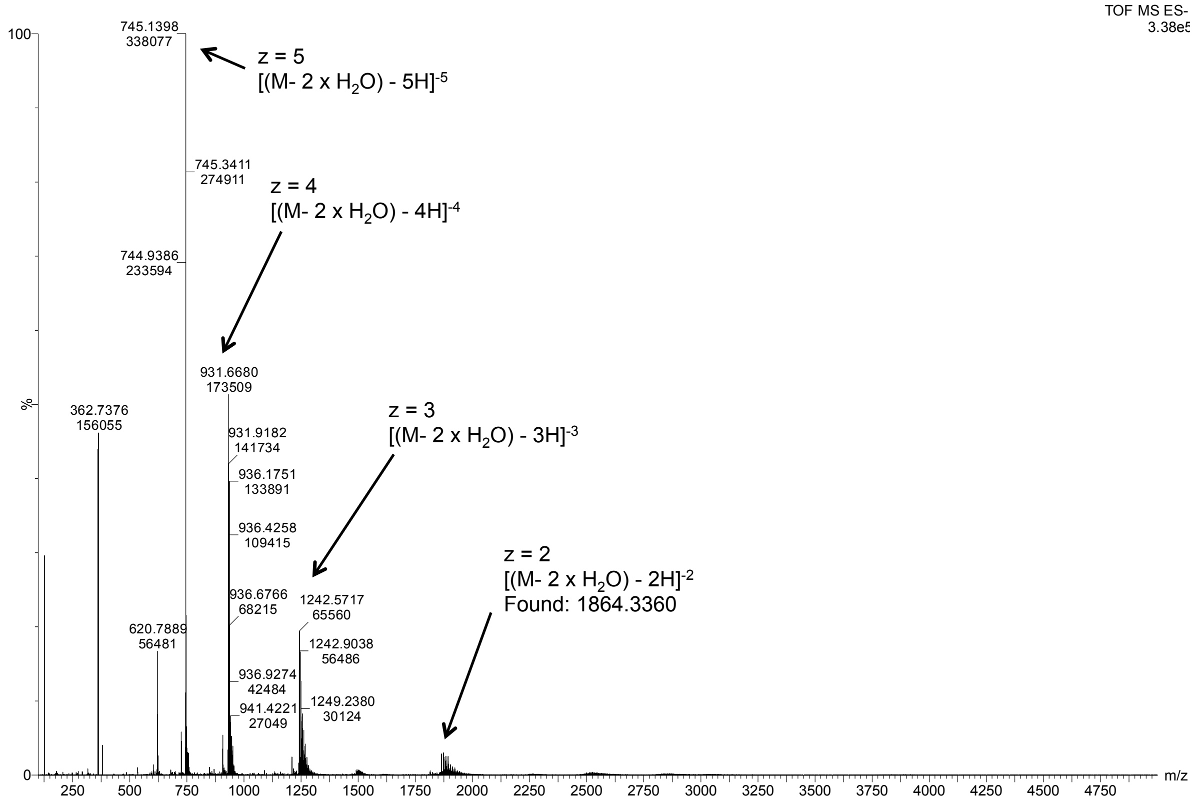

4-2. ON 14 5'-d(GCGTTT^{B}TTTCGT)-3' + H_2O_2

ON 14 was dissolved in 10 mM sodium phosphate buffer (pH 7.2) containing 100 mM NaCl to give a final strand concentrateon of 4.0 μM. To the **ON 14** solutions was added H_2O_2 (1 mM) and the resulting sample mixture was incubated for 30 min at room temperature in advance to the HPLC analysis and mass measurement.

HPLC

Column: Waters XBridge™ OST C18 2.5 μm, 4.6 x 50 mm

Gradient: 8-16%MeCN (over 30 min) in triethylammonium acetate butter (pH 7.0, 0.1 M)

Flow rate: 1.0 mL/min

Column temperature: 50 °C

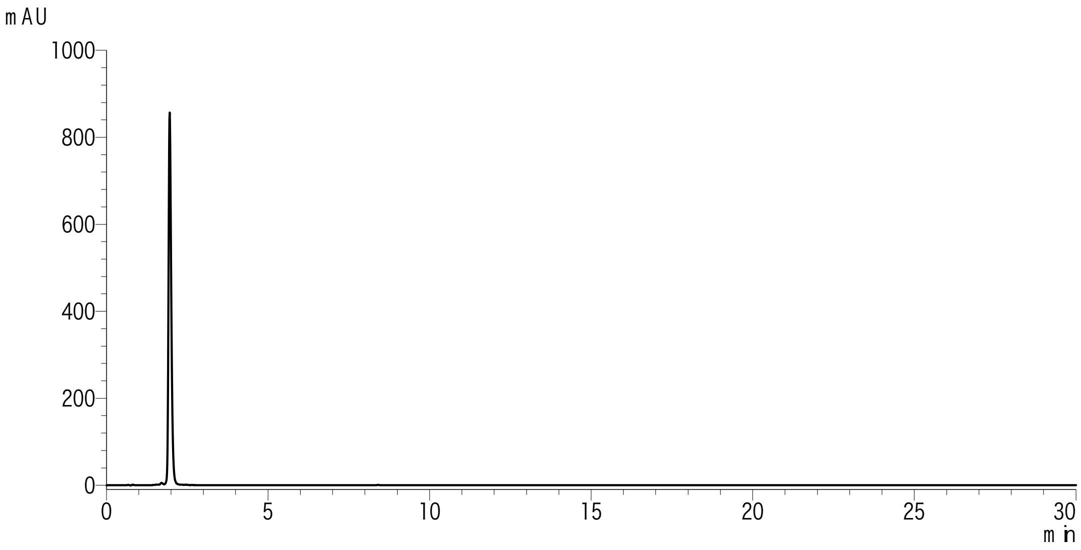

MALDI-TOF MS

Calcd. 3632.37 [M-H]$^{-}$

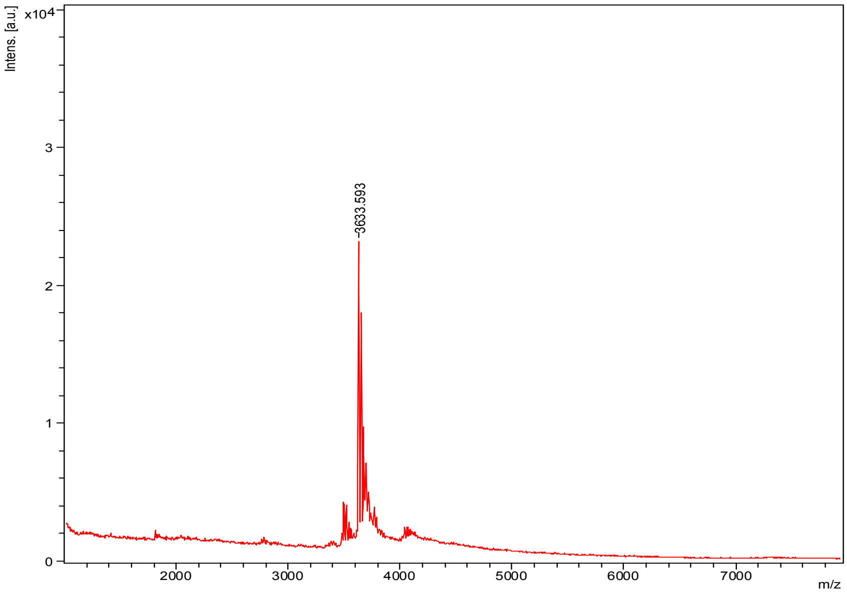

4-3. ASO S_0 5'-TC^{m}agtcatgactTC^m-3'

n = DNA N = LNA, all internucleosidic linkages are phosphorothioated

HPLC

Column: Waters XBridge^{TM} OST C18 2.5 μm, 4.6 x 50 mm

Gradient: 10-40%MeCN (over 30 min) in triethylammonium acetate butter (pH 7.0, 0.1 M)

Flow rate: 1.0 mL/min

Column temperature: 80 °C

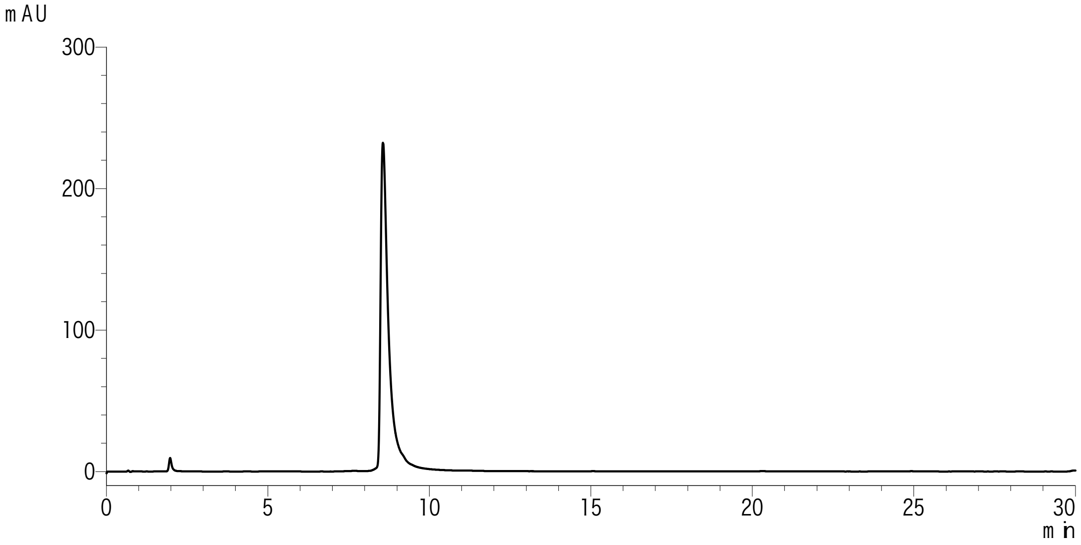

MALDI-TOF MS

Calcd. 4561.71 [M-H]$^-$

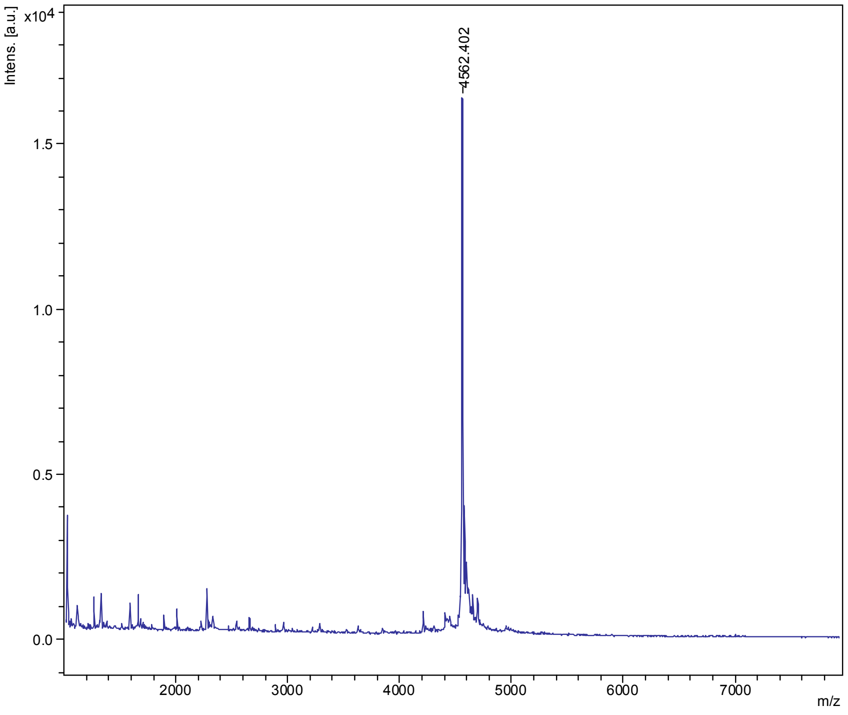

ESI

Calcd. 4562.71 [M]

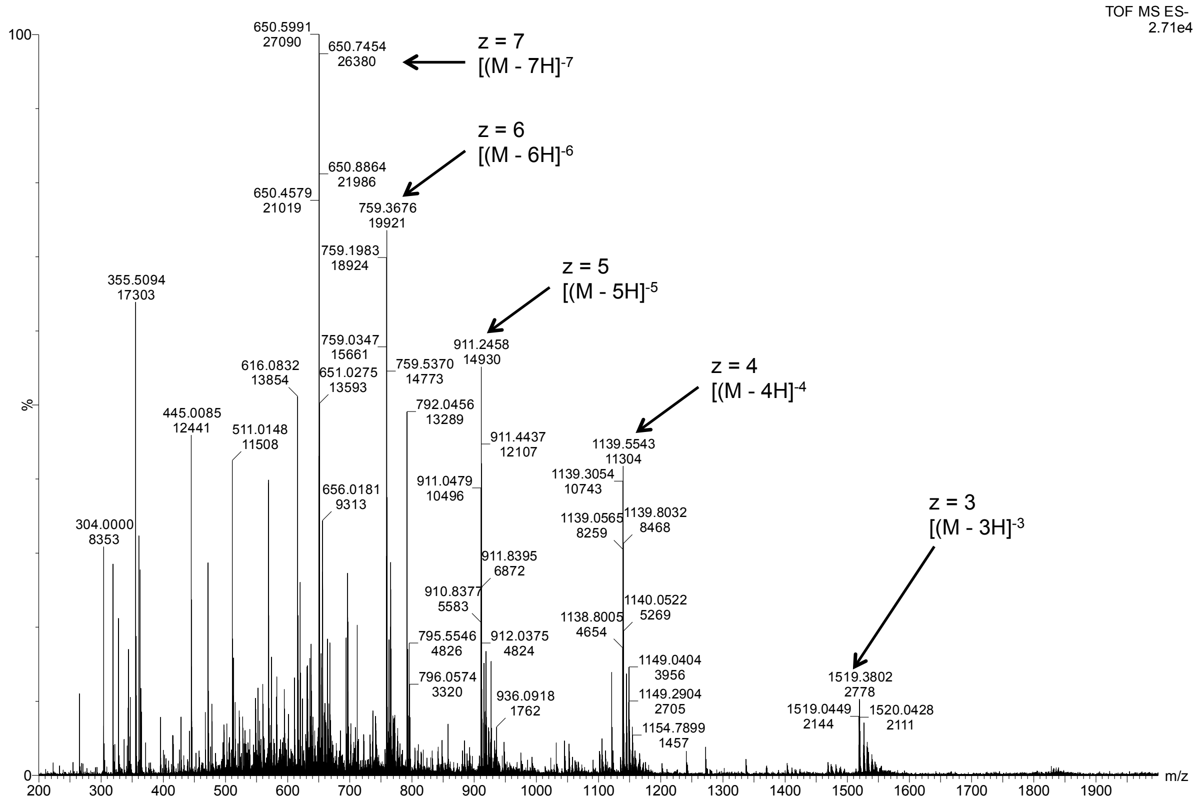

4-4. ASO S_1 5'-TC^{m}agt^{B}catgactTC^m-3'

n = DNA N = LNA, all internucleosidic linkages are phosphorothioated

HPLC

Column: Waters XBridge^{TM} OST C18 2.5 μm, 4.6 x 50 mm

Gradient: 10-40%MeCN (over 30 min) in triethylammonium acetate butter (pH 7.0, 0.1 M)

Flow rate: 1.0 mL/min

Column temperature: 80 °C

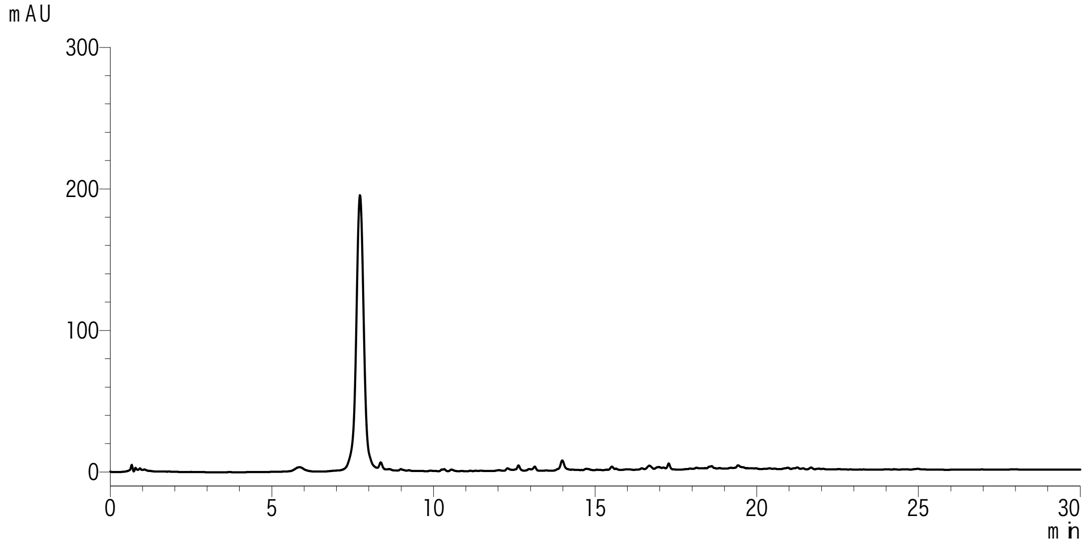

ESI MS

Calcd. 4660.62 [M - 2 x H_2O]

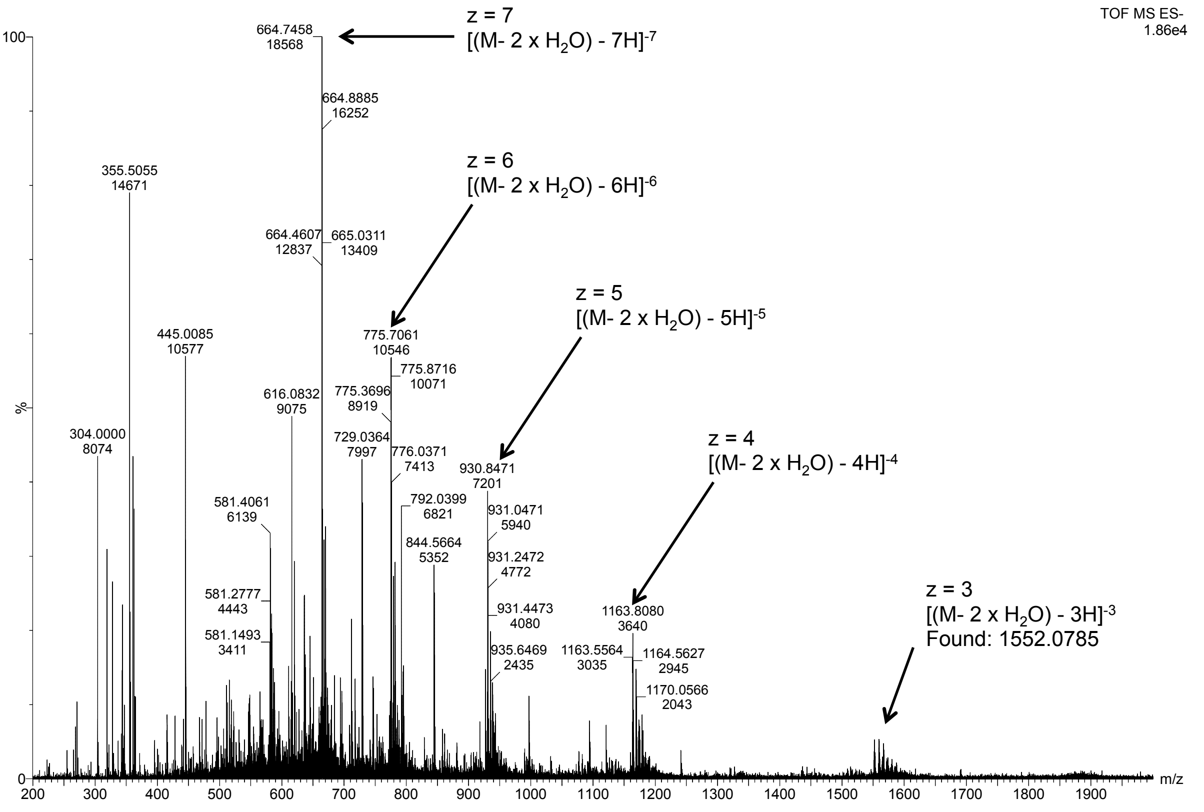

4-5. ASO S_2 5’-TC^{m}agt^{B}cat^{B}gactTC^m-3’

n = DNA N = LNA, all internucleosidic linkages are phosphorothioated

HPLC

Column: Waters XBridge^{TM} OST C18 2.5 μm, 4.6 x 50 mm

Gradient: 10-40%MeCN (over 30 min) in triethylammonium acetate butter (pH 7.0, 0.1 M)

Flow rate: 1.0 mL/min

Column temperature: 80 °C

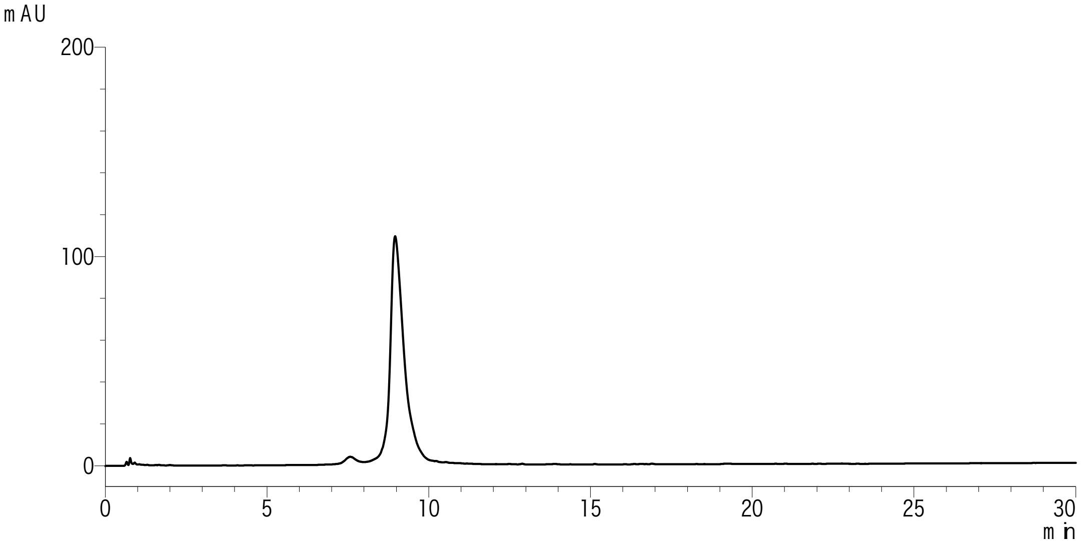

ESI MS

Calcd. 4758.52 [M - 4 x H_2O]

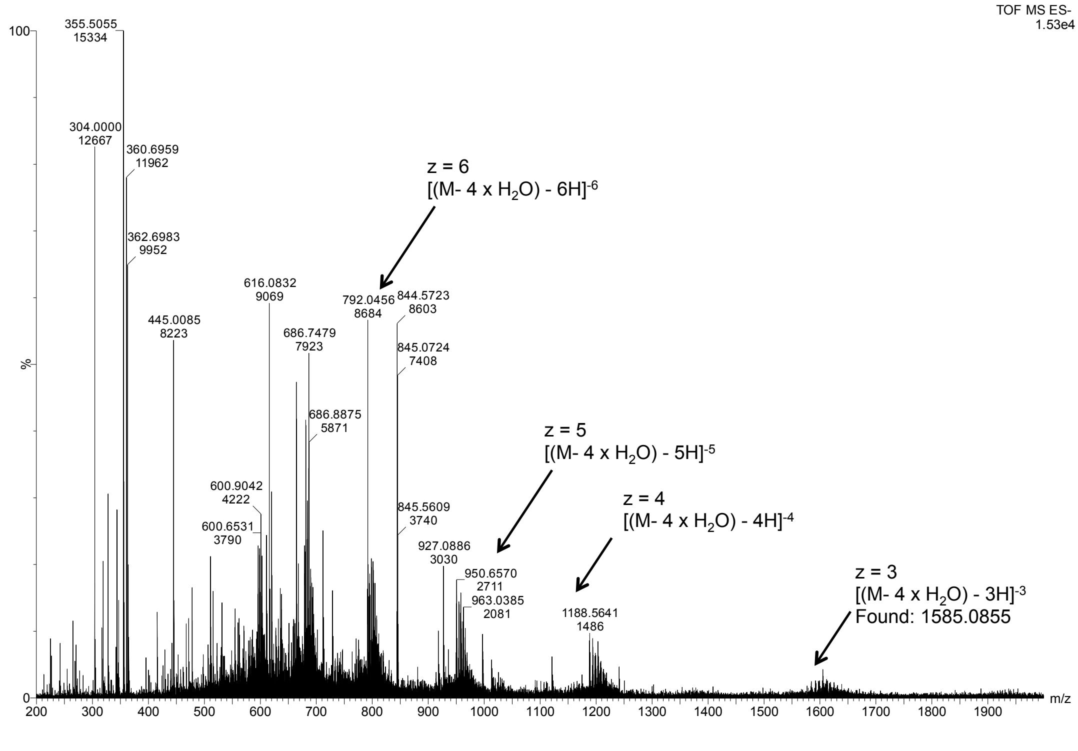

4-6. ASO S_3 5'-TC^{m}agt^{B}cat^{B}gact^{B}TC^m-3'

n = DNA N = LNA, all internucleosidic linkages are phosphorothioated

HPLC

Column: Waters XBridge^{TM} OST C18 2.5 μm, 4.6 x 50 mm

Gradient: 10-40%MeCN (over 30 min) in triethylammonium acetate butter (pH 7.0, 0.1 M)

Flow rate: 1.0 mL/min

Column temperature: 80 °C

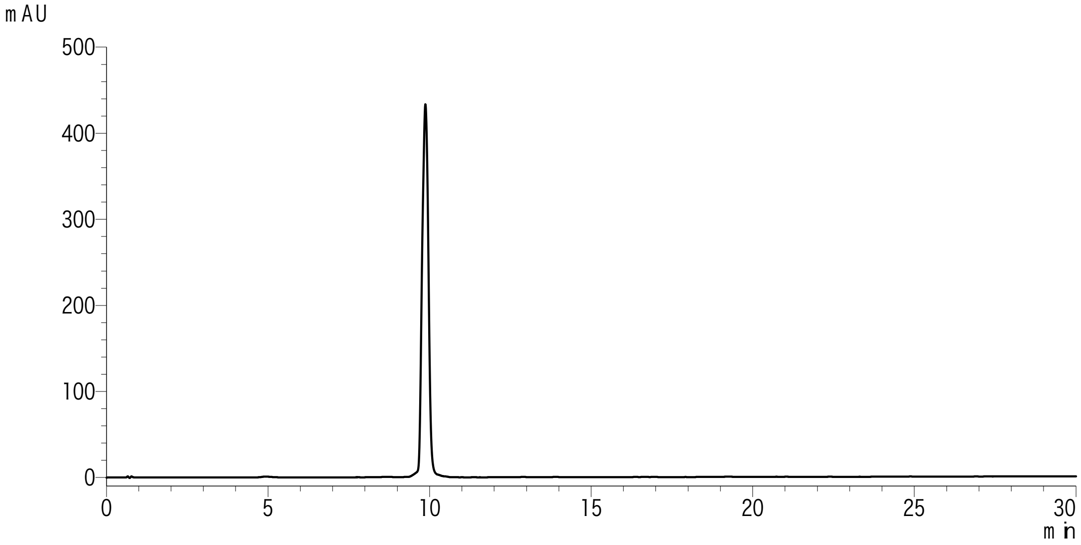

ESI MS

Calcd. 4892.45 [M - 4 x H_2O]

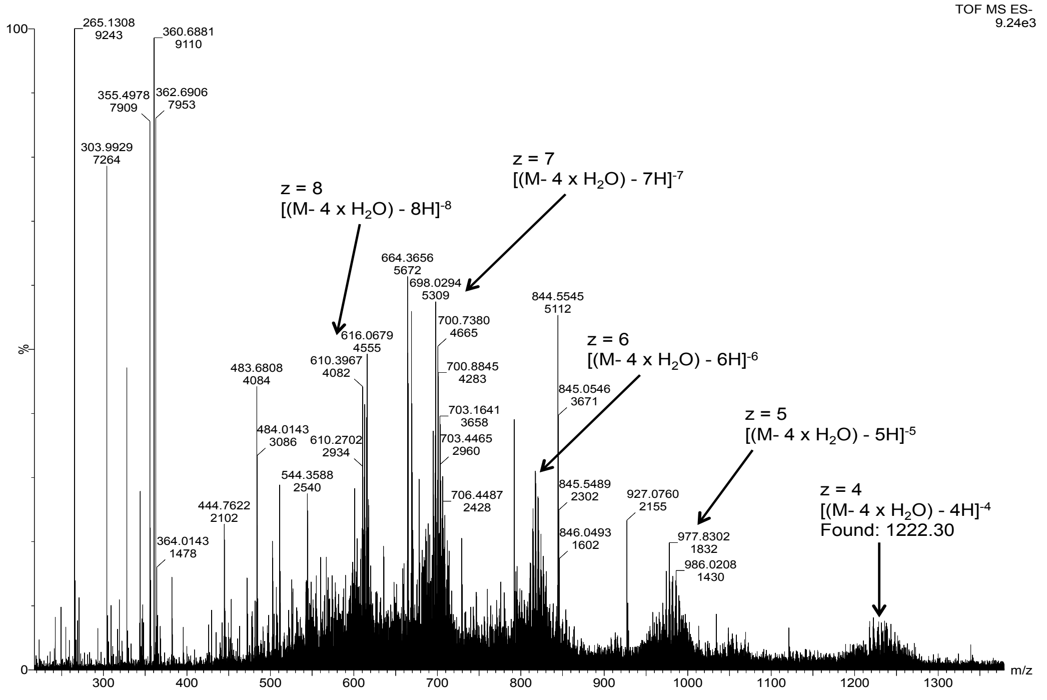

4-7. ASO S_A 5'-GC^{m}attggtatTC^{m}A-3'

n = DNA N = LNA, all internucleosidic linkages are phosphorothioated

HPLC

Column: Waters XBridge^{TM} OST C18 2.5 μm, 4.6 x 50 mm

Gradient: 10-40%MeCN (over 30 min) in triethylammonium acetate butter (pH 7.0, 0.1 M)

Flow rate: 1.0 mL/min

Column temperature: 80 °C

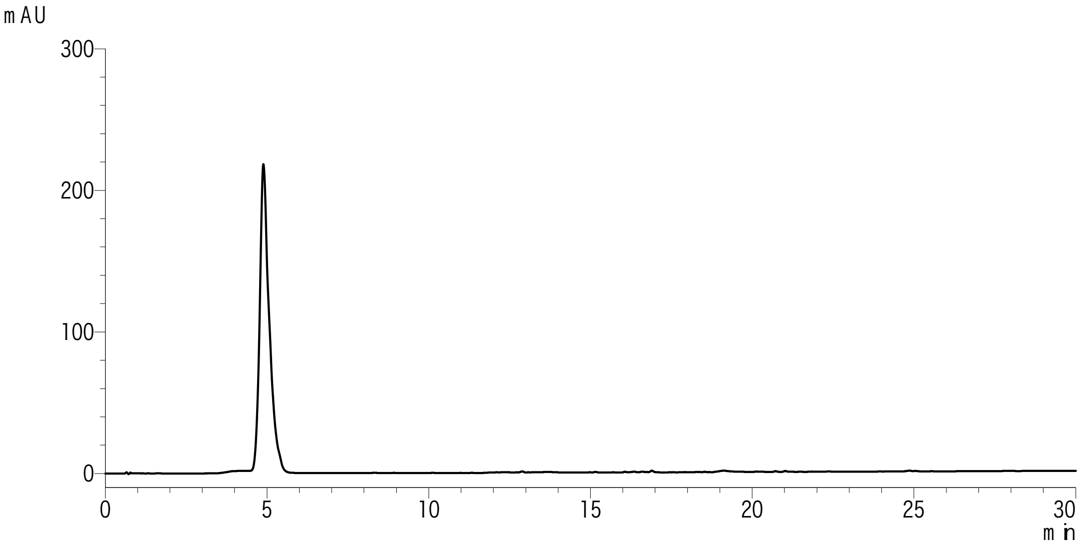

MALDI-TOF MS

Calcd. 4324.49 [M-H]$^-$

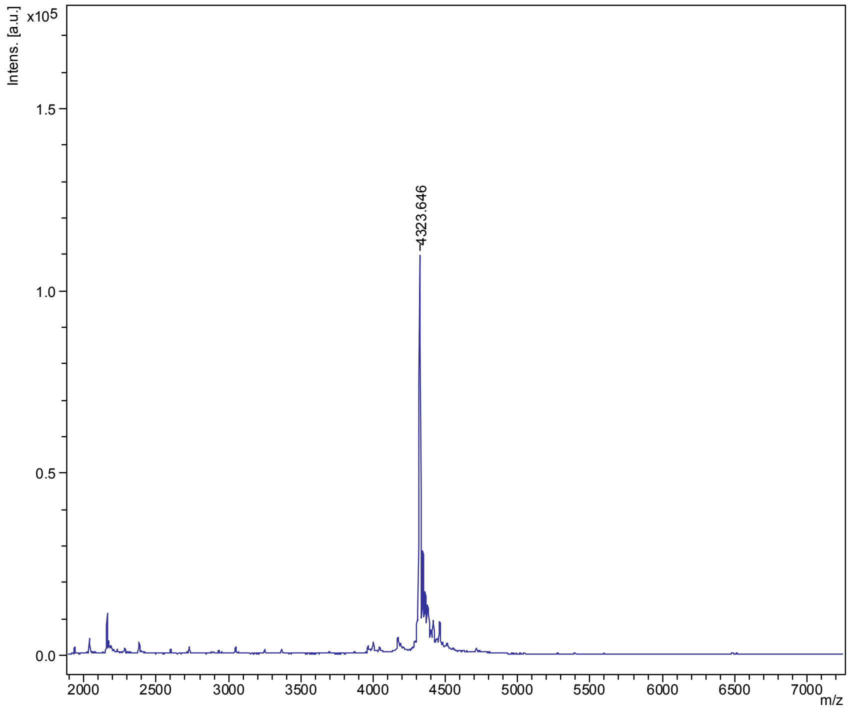

ESI MS

Calcd. 4325.49 [M]

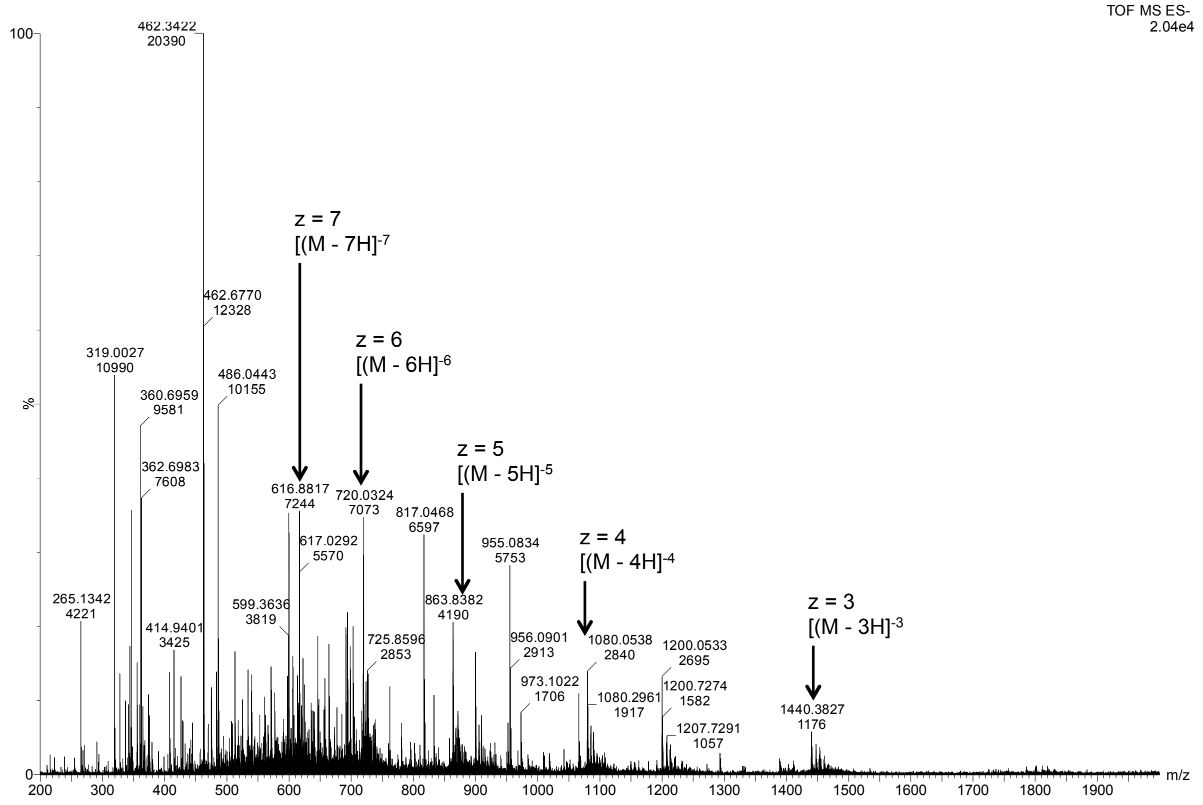

5. UV melting experiments of duplexes containing dT^B without or with H_2O_2

Equimolecular amounts of the target DNA/RNA and oligonucleotides were dissolved in 10 mM sodium phosphate buffer (pH 7.2) containing 100 mM NaCl to give a final strand concentration of 4.0 μM. Under the H_2O_2 presence condition, to the duplex solution was added H_2O_2 (1 mM) and the resulting sample mixture was incubated for 30 min at room temperature in advance to the UV melting experiments.

Table S1. Melting temperature of duplex between **ODN14** and RNA target in the presence or absence of H_2O_2.

ODN 5′–d(GCGTTXTTTCGT)–3′
cRNA 3′–r(CGCAAYAAAGCA)–5′

ODN	X	Y: A	G	C	U
		T_m/ °C			
14	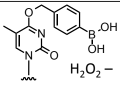 H_2O_2 −	32	39	30	31
14	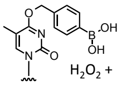 H_2O_2 +	46	39	30	32
15	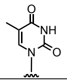	47	37	29	30

Table S2. Melting temperature of duplex between **ODN14** and DNA target in the presence or absence of H_2O_2.

ODN 5′–d(GCGTTXTTTCGT)–3′
cDNA 3′–d(CGCAAYAAAGCA)–5′

ODN	X	Y: A	G	C	U
		T_m/ °C			
14	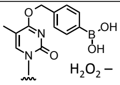 H_2O_2 −	31	35	33	34
14	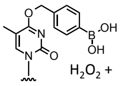 H_2O_2 +	52	41	36	38
15	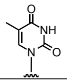	52	41	37	40

6. Reference

S1 C. Chung, D. Srikun, C. S. Lim, C. J. Chang and B. R.Cho, *Chem. Commun.*, 2011, **47**, 9618–9620.
